# Inhibition of *Cronobacter sakazakii* in an infant simulator of the human intestinal microbial ecosystem using a potential synbiotic

**DOI:** 10.3389/fmicb.2022.947624

**Published:** 2022-07-15

**Authors:** Alfred Ke, Valeria R. Parreira, Jeffrey M. Farber, Lawrence Goodridge

**Affiliations:** Canadian Research Institute for Food Safety, Department of Food Science, University of Guelph, Guelph, ON, Canada

**Keywords:** synbiotic, *Cronobacter sakazakii*, gut model, metabolomics, 16S sequencing

## Abstract

Powdered infant formula (PIF) can be contaminated with *Cronobacter sakazakii*, which can cause severe illnesses in infants. Synbiotics, a combination of probiotics and prebiotics, could act as an alternative control measure for *C. sakazakii* contamination in PIF and within the infant gut, but synbiotics have not been well studied for their ability to inhibit *C. sakazakii*. Using a Simulator of the Human Intestinal Microbial Ecosystem (SHIME^®^) inoculated with infant fecal matter, we demonstrated that a potential synbiotic, consisting of six lactic acid bacteria (LAB) strains and Vivinal GOS, can inhibit the growth of *C. sakazakii* in an infant possibly through either the production of antimicrobial metabolites like acetate, increasing species diversity within the SHIME compartments to compete for nutrients or a combination of mechanisms. Using a triple SHIME set-up, i.e., three identical SHIME compartments, the first SHIME (SHIME 1) was designated as the control SHIME in the absence of a treatment, whereas SHIME 2 and 3 were the treated SHIME over 2, 1-week treatment periods. The addition of the potential synbiotic (LAB + VGOS) resulted in a significant decrease in *C. sakazakii* levels within 1 week (*p* < 0.05), but in the absence of a treatment the significant decline took 2 weeks (*p* < 0.05), and the LAB treatment did not decrease *C. sakazakii* levels (*p* ≥ 0.05). The principal component analysis showed a distinction between metabolomic profiles for the control and LAB treatment, but similar profiles for the LAB + VGOS treatment. The addition of the potential synbiotic (LAB + VGOS) in the first treatment period slightly increased species diversity (*p* ≥ 0.05) compared to the control and LAB, which may have had an effect on the survival of *C. sakazakii* throughout the treatment period. Our results also revealed that the relative abundance of *Bifidobacterium* was negatively correlated with *Cronobacter* when no treatments were added (ρ = −0.96; *p* < 0.05). These findings suggest that *C. sakazakii* could be inhibited by the native gut microbiota, and inhibition can be accelerated by the potential synbiotic treatment.

## Introduction

Powdered infant formula (PIF) can be a vehicle of *Cronobacter sakazakii* infection in infants, which can cause the development of necrotizing enterocolitis (NEC), bacteremia, meningitis and neurological impairments, and the pathogen can be found in a wide variety of environments such as homes, hospitals, and manufacturing equipment (Kalyantanda et al., 2015; Centers for Disease Control and Prevention [CDC], 2020a,b). PIF contaminated with *C. sakazakii* poses a high risk of infection in infants, especially those that are pre-term (<37 weeks gestational age) and of low birth-weight (<2,500 g), and the consumption of PIF may not lead to the establishment of a diverse gut microbiota as compared to breast-fed infants (Le Doare et al., 2018). Despite recommendations from the World Health Organization (WHO) on best practices to rehydrate PIF, including using hot water (>70°C) to rehydrate PIF, *C. sakazakii* infections continue to occur and have resulted in a recent recall of PIF in addition to several hospitalizations and deaths in the United States and Canada (Centers for Disease Control and Prevention [CDC], 2022), so other mitigation strategies are needed. Probiotics, prebiotics, or a combination of both can be added to PIF to mimic the composition of breast milk (Ackerberg et al., 2012; Vandenplas et al., 2015), which is a challenge due to the various microbiota and oligosaccharides present.

A synbiotic is a combination of probiotics and prebiotic substrate(s) to confer a health benefit on the host (Swanson et al., 2020). Synbiotics can increase the populations of *Lactobacillus* and *Bifidobacterium* spp. in the gastrointestinal (GI) tract, improve immune compartment function and reduce the risk of bacterial infection in vulnerable patients (Pandey et al., 2015). However, interactions between the gut microbiota and probiotics can differ depending on the prebiotic. Therefore, compatibility between probiotics and prebiotic(s) is an important factor to consider when formulating synbiotics. The consumption of a synbiotic supplement in preterm infants can reduce the risk of infection by increasing the populations of beneficial gut microbiota, such as *Bifidobacteria*, and/or reducing the chances of pathogen adhesion to gut epithelial cells (Luoto et al., 2014; Kent et al., 2015), however, *in vivo* studies are rarely conducted due to ethical considerations. Furthermore, synbiotic supplementation in premature infants may have other benefits, including a decrease in NEC cases compared to the control group based on randomized clinical trials (Vongbhavit and Underwood, 2016).

An advanced approach to study *C. sakazakii* in the GI tract of humans is by using GI models to assess the pathogen’s survival and changes in the gut microbiota and metabolome under controlled conditions. As compared to *in vivo* animal models, GI models can better mimic the human intestinal environment, e.g., pH variation in the GI tract and colonic metabolome. The Simulator of the Human Intestinal Microbial Ecosystem (SHIME) is a versatile, multi-compartment human GI simulator that can mimic the conditions of the stomach, small intestine and colon (Van den Abbeele et al., 2010). Using the SHIME, it is possible to evaluate the interactions between the gut microbiota, gut metabolome and foodborne pathogens by inoculating the colon vessels with fecal samples from a donor and assessing different experimental parameters such as pH and residence time (Sivieri et al., 2011; Van de Wiele et al., 2015). Given the highly dynamic and naïve nature of an infant gut microbiota and its association with protecting the host from colonization by a foodborne pathogen, such as the production of antimicrobial compounds and preventing pathogen adhesion to the gut epithelial cells, the SHIME may be a useful tool to study the interactions between *C. sakazakii* and the gut microbiota metabolome in a simulated infant GI tract.

Probiotics, prebiotics, and synbiotics have the potential to play a major role in preventing intestinal colonization of infants by pathogenic *Cronobacter* spp. or other enteropathogens. However, there has not been any research on the effect of synbiotics on *C. sakazakii*, although several studies have shown that the ingestion of synbiotics can reduce the morbidity or mortality of NEC (Dilli et al., 2015; Sreenivasa et al., 2015; Nandhini et al., 2016; Pehlevan et al., 2020), and other studies have shown the antimicrobial properties of synbiotics on other foodborne pathogens such as *Salmonella enterica*, *Shigella sonnei*, and enteropathogenic and enterohemorrhagic *E. coli* (Likotrafiti et al., 2016; Kusmivati and Wahyuningsih, 2018; Shanmugasundaram et al., 2019; Tabashsum et al., 2019; Piatek et al., 2020). Probiotics, prebiotics and synbiotics can reduce the risk of infection through various mechanisms including competitive exclusion, the production of antimicrobial compounds, or competition for nutrients, but further research is required to validate these hypotheses using more sophisticated *in vitro* models and clinical trials. The development of comprehensive *in vitro* models that simulate the human gut, such as the SHIME, provides an interesting opportunity to study the real-time interactions between *C. sakazakii* and the native infant gut microbiota *in vitro*. Here, we demonstrate that the SHIME can provide valuable evidence on the survival of *C. sakazakii* with and without a potential synbiotic treatment through the perspective of culture-based and bioinformatic methods. We also show that the inhibition of *C. sakazakii* in an infant SHIME may be due to the presence of the gut microbiota, the production of metabolites or multiple factors.

## Materials and methods

### Preparation of fecal slurry for the infant simulator of the human intestinal microbial ecosystem

Fecal samples were collected from a healthy 1-month old infant, as very young infants (<2 months) represent the most vulnerable population to *C. sakazakii* The infant was of normal birth weight and gestational age, and was breast fed (but supplemented with formula) and had no history of sickness or antibiotic use. Fecal samples were collected in diapers at the donor’s residence and frozen. The samples were kept frozen using ice packs in a Styrofoam box during transportation to the Canadian Research Institute for Food Safety (Guelph, ON, Canada) and immediately stored at −80°C prior to fecal extraction from the diapers. Fecal samples were extracted from the diapers aseptically and transferred into 50 mL Falcon tubes. Both the tubes and the samples were kept frozen throughout the extraction process to minimize thawing and potential loss of microbes in the fecal samples. The tubes were weighed before and after to obtain the net weight of the fecal samples in each tube.

On the day of the SHIME inoculation, the fecal slurry was mixed with sterilized phosphate buffer (0.1 M; pH 7) containing: K_2_HP_*O*4_ (8.8; Alfa Aesar, Tewksbury, MA, United States), KH_2_P_*O*4_ (6.8; Anachemia Science, Mississauga, ON, Canada), sodium thioglycolate (0.1; Sigma-Aldrich, Oakville, ON, Canada). Prior to use, 15 mg of sodium dithionite (Sigma-Aldrich, Oakville, ON, Canada) was added to the buffer solution. The buffer solution was added to the partially thawed tubes containing fecal samples at a volume of 20% (w/v) and homogenized by pipetting up and down in the tube. After homogenization, the proximal and distal colon vessels were inoculated with 5% (v/v) of the fecal inoculum.

### Experimental design of the infant simulator of the human intestinal microbial ecosystem

This SHIME experiment was approved by the University of Guelph and conducted in accordance with the University guidelines and recommendations (REB# 20-12-004). The infant SHIME consisted of three parallel SHIME compartments. Each compartment was comprised of three double-jacketed glass vessels to simulate the stomach and small intestine (ST/SI), proximal colon, and distal colon and maintained at 37°C using a water bath. Anaerobic conditions were maintained by flushing all the SHIME vessels with nitrogen. The pH of each vessel representing the proximal and distal colon was monitored daily and adjusted to pH values of 6.0–6.2 and 6.0–6.5, respectively, using 0.5 M HCl and 0.5 M NaOH to simulate physiological conditions.

**FIGURE 1:**
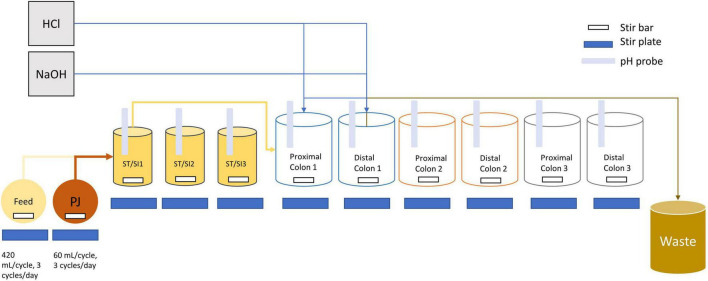
Schematic diagram of the infant triple SHIME. Tubing connections are only shown for SHIME 1 as an example, but SHIME 2 and 3 are connected in the same manner. Each SHIME compartment had one stomach/small intestine (ST/SI), proximal and distal colon vessel. Feed and contents from the proximal colon were transferred into each of the ST/SI vessels three times a day. Contents from the ST/SI vessels are then transferred into the proximal colon while contents from the proximal colon were transferred into the distal colon. HCl and NaOH were added into the vessels as needed to maintain a uniform pH range. Contents from the distal colon were transferred into a waste bucket for disposal. All vessels were kept at 37°C and consistently flushed with nitrogen to maintain an anaerobic environment. PJ refers to pancreatic juice.

The proximal and distal colon vessels contained basal feed at a volume of 300 and 500 mL, respectively. The basal feed (ProDigest, Belgium) contained the following (g/L): pectin (1), starch (1), cellobiose (1), proteose peptone (2), mucin (6), lactose (2.1), casein (0.2), whey proteins lactalbumin (2.7) and L-cysteine-HCl (0.2). Prior to use, the basal feed was acidified to a pH value of 3 using concentrated HCl. Pancreatic juice was made in addition to the basal feed based on the following composition (g/L): NaHCO3 (2.5; Anachemia Science, Mississauga, ON, Canada), Oxgall (4; Difco, Detroit, MI, United States), pancreatin from porcine pancreas (0.9; Sigma-Aldrich, Oakville, ON, Canada). A volume of 60 mL of pancreatic juice and 140 mL basal feed was transferred to the ST/SI vessels three times every day and mixed before being transferred through the SHIME tubes simultaneously (Figure 1). After fecal inoculation, a stabilization period, which lasted approximately 3 weeks, began, followed by the treatment period (Supplementary Figures 1, 2).

### Bacterial strains and culture conditions

Each SHIME compartment received two treatments spanning 1 week each, with SHIME 1 representing the control for the entire duration of the 2-week treatment period (Table 1). The lactic acid bacteria (LAB) and Vivinal GOS (VGOS), which is a commercial prebiotic substrate incorporated within infant formula, and used as the potential synbiotic in the infant SHIME, were formulated as previously described (Ke et al., 2022). The selected *C. sakazakii* and LAB strains (Supplementary Table 1) were grown in BHI or MRS broth, respectively, overnight at 37°C then diluted 1:100 the following day in fresh BHI or MRS broth and incubated for a further 24 h at 37°C. For the potential synbiotic, hereafter referred to as LAB + VGOS, the overnight cultures of LAB were centrifuged at 14,000 × *g* for 10 min at room temperature and resuspended in MRS, which did not contain any carbohydrates, containing 1% VGOS. The cultures, present at a level of approximately 9–10 log CFU/mL, were mixed in equal volumes (1:1) and inoculated at a volume of 1% (v/v) into the proximal colon vessels of each SHIME compartment to directly mimic the effects of the treatments on *C. sakazakii* in the large colon. Samples from the proximal and distal colon vessels were taken throughout the 2-week treatment period to enumerate *C. sakazakii* survival, metabolomic analysis and 16S rRNA gene sequencing. Samples were serially diluted in 0.1% PW (10^–1^ to 10^–6^), plated on Brilliance *C. sakazakii* agar (Oxoid, Nepean, ON, Canada) in duplicate, and incubated for 24–72 h at 37°C.

**TABLE 1:** Treatment periods for each SHIME compartment spanning approximately 1 week each.

SHIME	Treatments		

	First treatment period	Second treatment period
1	*C. sakazakii* (∼9 log CFU/mL)	Blank MRS^1^
2	*C. sakazakii* (∼9 log CFU/mL) and LABs (∼9–10 log CFU/mL)	LABs with 1% VGOS (∼9–10 log CFU/mL)
3	*C. sakazakii* (∼9 log CFU/mL) and LABs with 1% VGOS (∼9–10 log CFU/mL)	LABs (∼9–10 log CFU/mL)

*^1^De Man, Rogosa, and sharpe media.*

### Metabolomics

Metabolomic analysis of the triple SHIME was conducted using 1D ^1^H nuclear magnetic (NMR) spectroscopy. Samples were taken from the proximal and distal colon SHIME vessels and passed through a sterile 1 μm pore size syringe filter (Fisher Scientific, Mississauga, ON, Canada), followed by a second filtration with a 0.8/0.2 μm pore size filter (VWR, Mississauga, ON, Canada). The internal standard (4,4-dimethyl-4-silapentane-1-sulfonic acid (DSS-d6) and sodium azide in D_2_O, Chenomx Inc., Alberta, AB, Canada) was added to the final filtrate of each sample at a 1:10 (v/v) ratio to obtain a final concentration of 0.5 ± 0.005 mM DSS. Samples were scanned on a 600 MHz spectrometer at the Advanced Analysis Centre (University of Guelph, Guelph, ON, Canada) within 48 h of preparation. NMR spectra were analyzed using the Chenomx NMR Suite version 8.6 (Chenomx Inc., Alberta, AB, Canada) and exported to Microsoft Excel.

### 16S rRNA gene sequencing of the simulator of the human intestinal microbial ecosystem samples

Samples were taken from the SHIME and stored in a −80°C freezer prior to extraction for genomic DNA (gDNA). Thawed SHIME samples totaling 3 mL were collected and centrifuged at 14,000 × *g* for 2 min to pellet the bacterial cells and the supernatant was discarded. A volume of 1 mL of QIAGEN InhibitEx buffer and 0.2 g of 0.1 mm zirconia beads (Biospec Products Inc., Bartlesville, OK, United States) were added to the tubes containing the SHIME bacterial pellets and subjected to bead beating at 3,000 rpm for 4 min (Disruptor Genie, Scientific Industries Inc., NY, United States), followed by boiling for 15 min. The tubes were centrifuged at 14,000 × *g* for 2 min and the supernatant was used in subsequent extraction steps following the QiAamp Fast DNA Stool Mini Kit (Qiagen, Mississauga, ON, Canada) instructions with some modifications to the final elution step. DNA was eluted in 100 μL of warmed elution buffer and incubated for 5 min before centrifuging at 14,000 × *g* for 1 min. Eluted DNA was quantified and qualified using the Qubit (Thermofisher, Massachusetts, United States) and QIAxpert (Qiagen, Mississauga, ON, Canada), respectively. The gDNA was stored at −20°C for downstream applications.

PCR amplification targeting the V4 region of the 16S rRNA gene was performed using the F515 (5′-GTGCCAGCMGCCGCGGTAA-3′) and R806 (5′-GGA CTACHVGGGTWTCTAAT-3′) primers (Caporaso et al., 2011) in addition to Nextera XT Index v2 sequences (Illumina). The PCR reactions were conducted in a 96-well plate containing 2 μL of template DNA, 1 μL each forward and reverse primer sequences at 200 nM and 21 μL of Invitrogen Platinum PCR SuperMix High Fidelity (Fisher Scientific, Mississauga, ON, Canada) to make a total PCR reaction volume of 25 μL. Thermocycler conditions were as follows: an initial denaturation step at 94°C for 2 min; followed by 30 cycles of denaturation at 94°C for 30 s, annealing at 65°C for 30 s, elongation at 68°C for 30 s; and 10 cycles of the same parameters but with annealing at 55°C, with a final extension step at 68°C for 5 min. The annealing temperature comprised of a 0.3°C increment touch-down starting at 65°C for 30 cycles, followed by 55°C for 10 cycles. PCR products were purified using AMPure XP beads (Fisher Scientific, Mississauga, ON, Canada) according to the Illumina 16S metagenomic sequencing library preparation guide and quantified on the Qubit. Purified libraries were normalized to 2 nM for each library and 8 μL of each library was pooled for sequencing at the Genomics Facility in the Advanced Analysis Center, University of Guelph, Guelph, ON, Canada. Sequencing data from the Miseq were processed using the QIIME2 (v2021.8) pipeline and the DADA2 plugin (Callahan et al., 2016). Classification of operational taxonomical units (OTUs) to the genus level was conducted using the pre-trained SILVA 138 99% OTUs from 515F/806R region of sequences (Quast et al., 2012; Bokulich et al., 2018). Validation of the *Cronobacter* genus from the SILVA classifier was done by comparing amplicon sequences to the National Center for Biotechnology Information (NCBI) database using the nucleotide BLAST, and the resulting hits allowed for confirmation of the genus and approximate speciation of *C. sakazakii* according to their closest match (≥97% identity match).

### Statistical analysis

The results obtained from the proximal colon and distal colon samples were pooled together as two biological replicates for statistical analysis (*n* = 2). All statistical analyses were conducted using R (v4.0.4). Metabolites were analyzed based on the differences in metabolite profile between treatment periods for each SHIME compartment and combined were evaluated using a Principal Component Analysis (PCA) biplot based on the factoextra package on R (Kassambara, 2017). Correlations between metabolite concentration and *C. sakazakii* levels were evaluated using linear regression. Significant correlation between metabolite concentration and *C. sakazakii* levels were determined using a *p*-value of 0.05.

Sequencing results were analyzed for alpha- and beta-diversity on R using the phyloseq package (McMurdie and Holmes, 2013). The alpha-diversity metric was measured based on the Shannon and Gini-Simpson indices, which calculates the species diversity, considering species richness and evenness, within various metadata. Significant differences in alpha-diversity measures were calculated based on the non-parametric pairwise Wilcoxon Rank Sum test. The beta-diversity metric was calculated based on unweighted UniFrac and Bray-Curtis dissimilarity and visualized on a principal coordinate analysis (PCoA) using the phyloseq package. Significant differences between groups were calculated based on permutational multivariate analysis of variance (PERMANOVA) statistical tests. PERMANOVA was conducted using the adonis function (permutations = 10,000) on the vegan package (v2.5-7; Oksanen et al., 2020). Spearman’s rank correlation was used to calculate correlations and *p*-values between and within metabolites and the microbial community. *P*-values obtained from the Wilcoxon Rank Sum test, PERMANOVA and Spearman’s rank correlation were adjusted using the Benjamini-Hochberg false discovery rate.

## Results

### Survival of *Cronobacter sakazakii* in the infant simulator of the human intestinal microbial ecosystem

**FIGURE 2:**
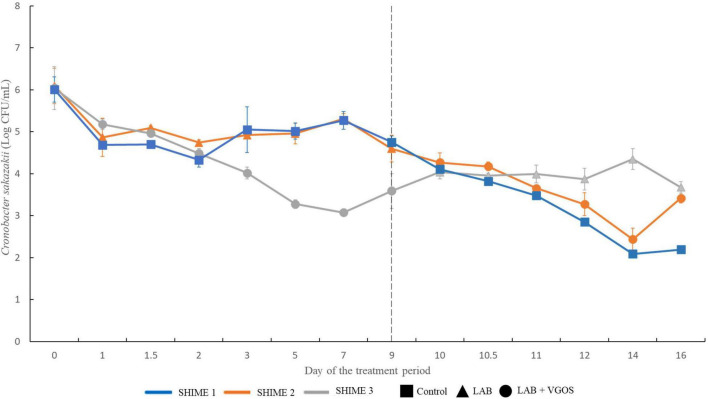
Survival of *C. sakazakii* in different SHIME compartments and under different treatment conditions (*n* = 2). At all time points, *C. sakazakii* was plated on Brilliance *C. sakazakii* agar. The gray dotted line indicates the start of the second treatment period. Values are the average of *C. sakazakii* plate counts from the proximal and distal colon vessels ± the standard deviation.

The change in *C. sakazakii* levels, based on the average of the plate counts from the proximal and distal colons of the SHIME, throughout the two treatment periods is shown in Figure 2. During the first treatment period and in the first 3 days, *C. sakazakii* decreased by 1–2 log CFU/mL for all SHIME compartments (*p* ≥ 0.05). Between 5 and 7 days, there was approximately 2-log CFU/mL difference in *C. sakazakii* levels between the SHIME containing the LAB + VGOS treatment and the other SHIME compartments (*p* < 0.05). However, there were similar *C. sakazakii* levels between the LAB-treated SHIME and the control (*p* ≥ 0.05).

During the second treatment period, there was no significant difference in *C. sakazakii* levels between all SHIME compartments between 9 and 12 days (*p* ≥ 0.05). At 14 days, there was a significant difference in *C. sakazakii* levels between the LAB-treated SHIME and the other SHIMEs compartments (*p* < 0.05). At 16 days, the *C. sakazakii* levels in the control SHIME were significantly different than the treated SHIMEs (*p* < 0.05).

Despite the change in treatments between two SHIME compartments, similar patterns were observed with regards to the reduction in numbers of *C. sakazakii*. Overall, *C. sakazakii* levels in the LAB-treated SHIME decreased by up to 1.5 log CFU/mL throughout the 1-week treatment period. In contrast, in the SHIME containing LAB + VGOS, the levels of *C. sakazakii* decreased by approximately 3 log CFU/mL after 5 to 7 days of the treatment (*p* < 0.05), and the control steadily decreased throughout the entire 2-week treatment period. Compared to the control and the LAB + VGOS-treated SHIME, the LAB-treated SHIME showed the least reduction in numbers of *C. sakazakii*.

### Metabolomic profile of the infant simulator of the human intestinal microbial ecosystem

**FIGURE 3:**
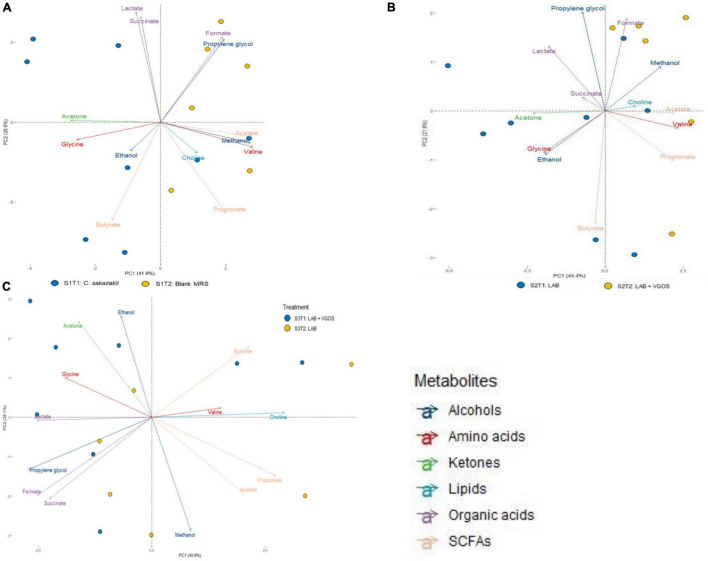
Principal Component Analysis (PCA) biplot of metabolite profiles from SHIME 1 **(A)**, SHIME 2 **(B)**, and SHIME 3 **(C)** over a 2-week treatment period. Each sample (bubble) represents a different day during the treatment period. The 2-week treatment period is separated with approximately 1 week for each treatment period and labeled accordingly based on color (i.e., blue for the first treatment period and yellow for the second). MRS represents de Man, Rogosa and Sharpe media, LAB represents lactic acid bacteria, VGOS represents Vivinal GOS, and SCFA represents short-chain fatty acids. Arrows represent the different metabolites profiled and colored based on the type of metabolite.

**FIGURE 4:**
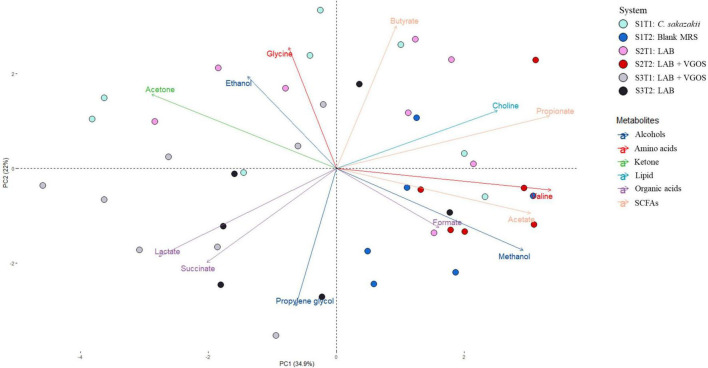
Principal Component Analysis (PCA) biplot of metabolite profiles from all SHIMEs across two treatment periods. The labels indicate the SHIME and treatment period. For example, S1T1 indicates that the light blue samples are for the first treatment period of SHIME 1. Each SHIME is represented by a different color and treatment periods are differentiated by the shade of the color, i.e., light or dark shade.

A principal component analysis (PCA) revealed the general relationship between metabolites and treatment periods for each SHIME (Figures 3, 4). The metabolite profile between the first and second treatment periods for SHIME 1 and 2 were different, but the profiles of SHIME 3 were similar (Figure 3). Based on the metabolite profiles of the SHIMEs, Principal Component 1 (PC1), accounting for 40.8% of the total variability of the data, may be associated between treatment periods as indicated with SHIME 1 and 2, and PC2, which accounts for 28.1% of the variability, may be associated with the metabolite concentrations.

The PCA biplot combining all SHIMEs and both treatment periods (Figure 4) also gave a different perspective on the overall relationship between SHIME compartments and treatment periods. In the first treatment period, the metabolite profiles of SHIME 1 and 2 (S1T1 and S2T1) were distinct from SHIME 3 (S3T1). A similar observation was observed in the second treatment period, as S3T2 was distinctly different than S1T2 and S2T2. Although we found a similar profile between treatment periods for SHIME 3, distinct metabolite profiles were found between treatment periods for SHIME 1 and 2. The PCA also indicated that the timing of the treatments may have had an effect on the metabolite profiles. S2T1 and S3T2, for example, were inoculated with the same types of LAB in similar concentrations, but yet the metabolite profiles for the two periods were distinct. When observed throughout the 2-week treatment period, however, no distinct metabolite profile was observed between the control and treatments (Supplementary Figure 3). During the first treatment period for SHIME 1 and 2, the change in *C. sakazakii* levels was similar (*p* ≥ 0.05; Figure 2). In the PCA, this was also indicated based on the similar metabolic profile observed for S1T1 and S2T1 (Figure 4). In contrast, SHIME 3 had reduced levels of *C. sakazakii* in the first treatment period compared to SHIME 1 and 2, which was shown by the distinct metabolite profile of S3T1 on the PCA compared to S1T1 and S2T1. This observation continued into the second treatment period, as samples from SHIME 1 and 2 had similar levels of *C. sakazakii* and similar clusters in the PCA.

**FIGURE 5:**
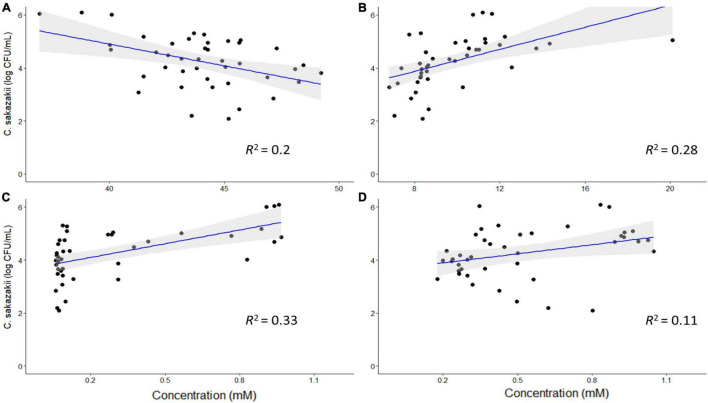
Regression analysis of *C. sakazakii* levels with concentrations (mM) of acetate **(A)**, ethanol **(B),** acetone **(C)**, and glycine **(D)**. There is a significant correlation between the concentrations of these metabolites and *C. sakazakii* levels (*p* < 0.05). Acetate is negatively correlated with *C. sakazakii*, whereas the other metabolites are positively correlated.

Based on the PCA analysis, some metabolites may be correlated with numbers of *C. sakazakii*, which were validated using a regression analysis. Acetate, ethanol, acetone and glycine concentrations were significantly correlated with *C. sakazakii* levels (*p* < 0.05; Figure 5). Acetate was negatively correlated with *C. sakazakii*, whereas the other metabolites were positively correlated. All of the other metabolites were not significantly correlated with *C. sakazakii* levels (*p* ≥ 0.05; Supplementary Figure 4).

### Microbial community compositions in the infant simulator of the human intestinal microbial ecosystem

**FIGURE 6:**
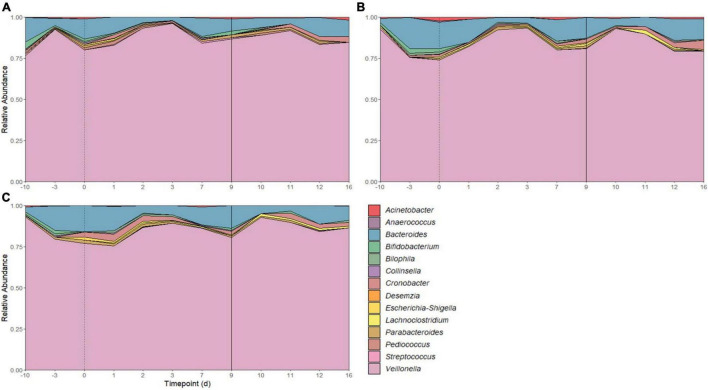
Microbial community composition of SHIME 1 **(A)**, SHIME 2 **(B)**, and SHIME 3 **(C)** before and throughout the treatment period. Microbial composition shown as average relative abundance and colored by genera. Relative abundances were based on 16S rRNA gene sequencing of SHIME samples from the distal colon vessels. Days –10 and –3 indicate the time points before the start of the first treatment period, as shown by a dotted line on day 0. Day 9 indicates the start of the second treatment period (solid line).

**FIGURE 7:**
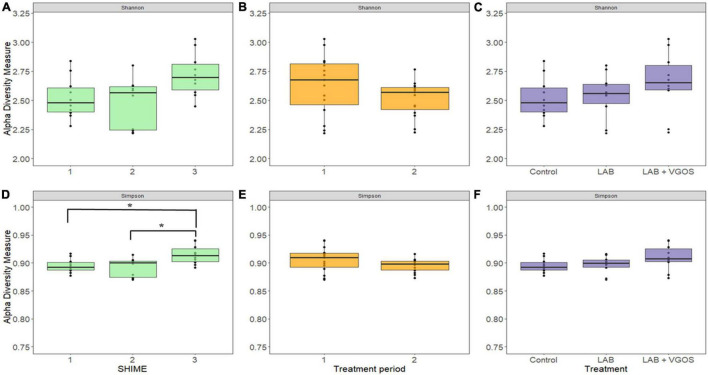
Shannon (top) and Gini-Simpson (bottom) indices for measurement of alpha diversity between SHIMEs **(A,D)**, treatment periods **(B,E)**, and treatments **(C,F)**. Significant differences in alpha diversity, as denoted by the asterisks in **(D)**, were calculated based on the Wilcoxon Rank Sum test and adjusted using the Benjamini-Hochberg false discovery rate (*p* < 0.05).

There were 14 shared genera within 5 phyla identified across all SHIME compartments. Firmicutes and Bacteroidetes were the dominant phyla in all SHIMEs (Supplementary Figure 5), and within these phyla, *Veillonella* and *Bacteroides* were the most abundant genera accounting for approximately 80–90% of the SHIME gut microorganisms (Figure 6). The species diversity across the three SHIME compartments remained fairly stable regardless of the treatment added. There was a slightly higher species diversity in the first treatment period across all SHIME compartments and during the LAB + VGOS treatment regardless of the SHIME compartment (*p* ≥ 0.05; Figure 7). The first inoculation of the LAB + VGOS treatment into SHIME 3 also showed a slightly higher species diversity relative to the other SHIMEs and treatment periods (*p* ≥ 0.05; Supplementary Figure 6). However, relative to the other SHIME compartments, SHIME 3 had the most diverse microbial community over the 2-week treatment period (*p* < 0.05; Figure 7). Furthermore, the presence of *C. sakazakii* did not alter the composition of the SHIME microbiota, even within the control SHIME in the absence of a treatment (*p* ≥ 0.05; Supplementary Figure 6).

**FIGURE 8:**
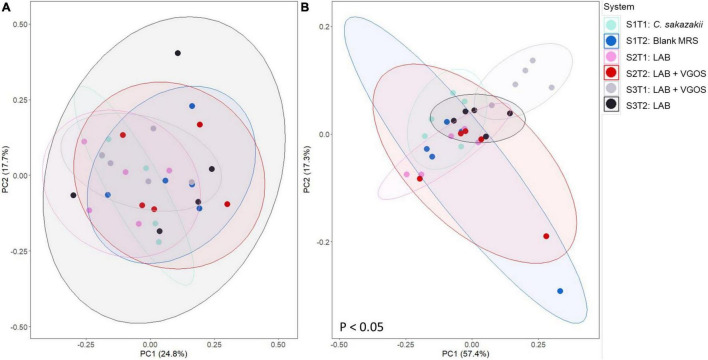
Principal coordinate analyses (PCoA) plots of unweighted UniFrac **(A)** and Bray-Curtis dissimilarity **(B)** based on operational taxonomical units from SHIME samples as visualized by treatment periods and SHIME, with ellipses indicating 80% confidence interval. The labels indicate the SHIME and treatment period. For example, S1T1 indicates that the light blue samples are for the first treatment period of SHIME 1. Each SHIME is represented by a different color and treatment periods are differentiated by the shade of the color, i.e., light or dark shade. Significant differences in groups were calculated based on PERMANOVA and adjusted using the Benjamini-Hochberg false discovery rate (*p* < 0.05).

Beta-diversity metrics (i.e., unweighted UniFrac and Bray-Curtis dissimilarity) showed significantly different shared taxa during treatment periods (*p* < 0.05; Supplementary Figure 7). Furthermore, community abundance was significantly different between SHIME, treatments, and both SHIME and treatments (*p* < 0.05; Figure 8 and Supplementary Figures 8, 9). Sub-setting of the metadata revealed that the difference in community abundance was due to SHIME 3 across both treatment periods and the LAB + VGOS treatment between treatments (*p* < 0.05).

### Metabolomic and gut microbiota associations in the simulator of the human intestinal microbial ecosystem

**FIGURE 9:**
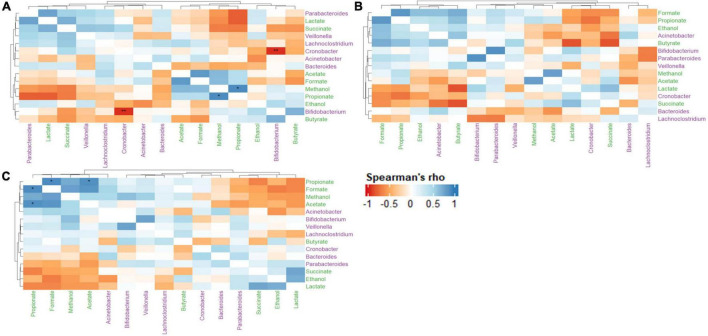
Heatmap of Spearman’s rank correlation coefficients of select metabolites and microbial genera between the control **(A)**, LAB **(B)**, and LAB + VGOS **(C)** treatments. Microbial genera are colored in green, and metabolites are colored in purple. A positive correlation indicates that when a microbial genera or metabolite becomes more or less abundant, so does the other feature. Conversely, a negative correlation indicates that when a genera or metabolite becomes less abundant, the other feature increases in abundance. Statistical significance is indicated by the asterisks after correction with Benjamini-Hochberg false discovery rate, *P < 0.05, **P < 0.01.

The relationships between metabolite-metabolite, metabolite-microbe and microbe-microbe pairs between the control and treatments (LAB and LAB + VGOS) were assessed using the Spearman’s rank correlation to provide some insight into the *C. sakazakii* survival, metabolomics and gut microbiota trends as previously described. In general, there were similar positive correlations between formate, propionate, ethanol, methanol, and acetate between the treatments and the control (Figure 9). There was also a stronger metabolite-metabolite correlation in the LAB + VGOS treatment as compared to the LAB only treatment. In particular, there were strong correlations between propionate and acetate, formate and methanol (ρ = > 0.73; *p* < 0.05), which suggests that these metabolites were part of similar metabolic pathways that may have been strengthened due to the LAB + VGOS treatment. Other trends in metabolite-metabolite correlation between different groups of metabolites were observed to be unique within the LAB + VGOS treatment, such as the negative correlation of the SCFAs with other organic acids, e.g., propionate with succinate and lactate, and the positive correlation between organic acids and ethanol. Between the metabolite-microbe and microbe-microbe pairs, the relative abundance of *Bifidobacterium* and *Cronobacter* in the control SHIME was negatively correlated (ρ = −0.96; *p* < 0.05), which may indicate a potential role of *Bifidobacterium* in controlling *Cronobacter* in a healthy infant.

## Discussion

*Cronobacter sakazakii* can pose a significant risk to infants when ingested, but the risk of illness may be reduced by ingesting probiotics or synbiotics which could inhibit the growth of *C. sakazakii*. Here, we demonstrated that a potential synbiotic consisting of LAB and VGOS can inhibit the growth of *C. sakazakii* in an infant SHIME model, but *C. sakazakii* levels also decreased, albeit slower, in the absence of a treatment. Metabolomics and 16S rRNA gene sequencing showed potential mechanisms of inhibition, such as the production of antimicrobial metabolites, or possibly through competition for nutrients due to a more diverse microbial community as a result of the potential synbiotic treatment.

In the infant SHIME, *C. sakazakii* levels significantly decreased after both applications of LAB + VGOS treatment and throughout the 2-week treatment period in the control SHIME. In contrast, both applications of the LAB treatment did not seem to have a significant effect on *C. sakazakii* levels regardless of the SHIME compartment. Synbiotics can increase *Lactobacillus* and *Bifidobacterium* levels and were found to be better at inhibiting foodborne pathogens than probiotics, as prebiotic substrates can be degraded by the probiotics or the gut microbiota into metabolites that could have antimicrobial properties (Likotrafiti et al., 2016; Markowiak and Śliżewska, 2017; Piatek et al., 2020; Sakr and Massoud, 2021).

The earliest colonizers of the infant gut are LAB and bifidobacteria, although the latter is generally more predominant (Martín et al., 2003; Solís et al., 2010). The numbers of LAB in infant feces range from 6 to 10 log CFU/g (Rubio et al., 2014; Murphy et al., 2017), which is in similar quantities to the concentration of LAB (9–10 log CFU/mL) used in the potential probiotic and synbiotic treatments. The LAB used in this study were not tested for acid or bile resistance, therefore their independent introduction into the SHIME may have reduced their survival rate as they were exposed to gastric and bile juices and other bacteria.

Probiotics and prebiotics, and by extension synbiotics, can influence the gut metabolome by modulating the gut microbiota (Abdulkadir et al., 2016; Chung et al., 2018; O’Connell, 2020). As a result, metabolomics, alone or in combination with 16S rRNA gene sequencing, can be used to evaluate changes in host health. In particular, short-chain fatty acids (SCFA) are regarded as some of the most important metabolites produced in the GI tract due to their association with host health (Koh et al., 2016; LeBlanc et al., 2017). Some of the key functions of SCFA are serving as energy sources for epithelial cells, inhibiting opportunistic pathogens and modulating the immune compartment (Koh et al., 2016).

In this study, metabolomic analyses revealed that the application of the LAB and LAB + VGOS in an infant SHIME affected the infant gut metabolome in a time-dependent manner. In general, there seems to be a time-dependent association between metabolomic profiles and treatments. Probiotics can influence the levels of SCFA and other metabolites, but the effects might be specific to the individual (Ghini et al., 2020; Markowiak-Kopeć and Śliżewska, 2020). Previous studies have also reported that metabolomic profiles are more stable than microbiota profiles (Abdulkadir et al., 2016; O’Connell, 2020), which may indicate that the beneficial effects of probiotics may not be revealed solely by metabolomics analyses. The infant diet, which includes prebiotics and breast milk, can alter the metabolomic profile of infants by shifting the composition of their gut microbiota (Stewart et al., 2017; Brink et al., 2020). A synbiotic consisting of *Lactobacillus paracasei* with GOS was found to increase the growth of lactobacilli and bifidobacteria, which subsequently increased the concentration of acetate in gnotobiotic mice inoculated with infant microbiota, as compared to the control (Martin et al., 2010). Synbiotic supplementation can also positively impact gut metabolic activities, as indicated by an increase in levels of SCFA, ketones, carbon disulfide and methyl acetate (Vitali et al., 2010). It is worthwhile to note that even in healthy, breast-fed infants, the metabolic profiles between infants can vary (Kirchberg et al., 2020), although this may be partially due to limitations in the studies’ methodologies and analyses (Phan et al., 2019).

Organic acids and bacteriocins are produced by probiotics and synbiotics that can inhibit the growth of pathogens (Tomar et al., 2015; Markowiak and Śliżewska, 2017). In the infant SHIME, the pH is controlled to be between 6.0–6.2 and 6.0–6.5 for the proximal and distal colon vessels, respectively, which may have an effect on the ability of the organic acids to inhibit the growth of *C. sakazakii*. Likotrafiti et al. (2016) showed that the combination of *B. longum* or *L. fermentum* 907 and various prebiotics inhibited the growth of *E. coli* O157:H7 and *E. coli* O86 when the pH of the media was lower than the pKa values of the organic acids. The authors noted that the difference in pH of the media with *B. longum* containing either glucose or VGOS was a factor in whether the pathogens’ growth was delayed or inhibited. Weak acids like organic acids can enter a bacterial cell through the cell membrane and acidify the cytoplasm, which in turn can damage enzymes and cell structures (Gao et al., 2019). Furthermore, organic acids can play a role in stabilizing the microbial population in the gut and stimulating the growth of commensal bacteria (Dittoe et al., 2018). Bacteriocins, unlike organic acids, are polypeptides produced by bacteria during ribosomal synthesis and can also have antimicrobial properties (Gao et al., 2019). Some LAB used in this study, e.g., *L. paracasei* and *P. acidilactici*, have been shown to produce bacteriocins (Papagianni and Anastasiadou, 2009; Bendjeddou et al., 2012), but the particular strains used in this study have not been tested for bacteriocin production. Furthermore, due to the lack of an outer membrane, bacteriocins are more effective against Gram-positive than Gram-negative bacteria (Prudêncio et al., 2015; Simons et al., 2020), so they are unlikely to have been the causative factor that inhibited *C. sakazakii*.

Previous studies have shown that hydrogen peroxide (H_2_O_2_) is produced by LAB and can inhibit the growth of foodborne pathogens through oxidative stress (Tharrington and Sorrells, 1992; Kotsou et al., 2008). In the anaerobic conditions of the gut and the infant SHIME, the concentration of H_2_O_2_ may not be enough to inhibit the growth of pathogens (Knaus et al., 2017). Additionally, sub-lethal concentrations of H_2_O_2_ can increase the resistance of foodborne pathogens and their progeny (Rodríguez-Rojas et al., 2020), however, whether this occurs in the SHIME ecosystem is unclear.

An alternative mechanism by which probiotics and synbiotics inhibit foodborne pathogens is by competing for limited nutrients in the environment, which suggests that beneficial bacteria in the gut can use nutrients that would otherwise be used by opportunistic pathogens, thereby limiting their growth (Rolfe, 2000). There are more nutrients available in the proximal colon, as compared to the distal colon, due to its proximity to the stomach and small intestine, so increasing the levels of beneficial bacteria in the proximal colon can decrease nutrient availability for pathogens (Fooks and Gibson, 2002). Wilson and Perini (1988) found that fecal bacteria used glucose and other nutrients more efficiently than *Clostridium difficile*, which reduced its growth compared to the control without fecal bacteria. Furthermore, even bacteria within the same genera may compete for nutrients with opportunistic pathogens and each other as they have similar metabolic requirements (Fleming-Davies et al., 2017; Patnode et al., 2019). In contrast, depleted gut microbiota as a result of antibiotic treatment can lead to an increase in the amount of nutrients in the gut environment and limiting competition for nutrients with commensal gut microbiota, which can cause the proliferation of *Salmonella* Typhimurium and *C. difficile* (Ng et al., 2013). Similarly, Gillis et al. (2018) found that dysbiosis can cause the host metabolism to undergo lactate fermentation, which in turn facilitates the growth of *Salmonella*.

Metabolomics analyses gave some insight into the possible factors that could inhibit *C. sakazakii*. Untargeted metabolomics and PCA have been used to screen for potential bioactive molecules from plants and evaluate the metabolic changes of foodborne pathogens post-exposure to biochemical or physical treatments (Maifiah et al., 2017; Tian et al., 2018; Sieniawska and Georgiev, 2022). In this study, the PCA biplot revealed differences in metabolite profile within the SHIME compartments that may be generally associated with *C. sakazakii* levels. Additionally, there was no observed difference in metabolite profiles when comparing between the treatments and the control. Maifiah et al. (2017) used PCA to identify changes and similarities in the metabolite profile of *Acinetobacter baumannii* after antibiotic treatment at different time points. Additionally, Tian et al. (2018) used PCA to identify changes in the metabolomic response of *E. coli* after exposure to ohmic heating. This study found that some metabolites, such as valine, alcohols, acetate and other organic acids are generally associated with lower levels of *C. sakazakii.*

Regression analysis of the profiled metabolites revealed that acetate, ethanol, acetone and glycine were significantly correlated with *C. sakazakii* levels. However, of these metabolites, only acetate was negatively correlated with *C. sakazakii.* Acetate, in addition to propionate and butyrate, has been reported to reduce the growth and pathogenicity of foodborne pathogens (Peng and Biswas, 2017; Zhang et al., 2020). In this study, however, only acetate was shown to be a significant factor in reducing the levels of *C. sakazakii*. The efficacy of acetate as an antimicrobial compound could be affected by pH, where a lower pH would allow SCFAs and other organic acids to permeate bacterial cells and acidify the cytoplasm (Lawhon et al., 2002; Lund et al., 2020). For example, Zhang et al. (2020) reported that SCFAs in a broth medium with a pH of 6.5 were able to inhibit the growth of *E. coli*, whereas a neutral pH stimulated its growth. Foodborne pathogens, including *C. sakazakii*, can develop acid resistance when exposed to sub-lethal concentrations of acetate, although the adaptation is influenced by the acid concentration and the pH of the environment (Arnold et al., 2001; Álvarez-Ordóñez et al., 2012; Gavriil et al., 2020). *E. coli* can adapt to the acid stress induced by SCFAs at a neutral pH by regulating the expression of *rpoS* (Arnold et al., 2001). Changes in protein synthesis have been linked to an increase in resistance to salt and acid (pH value of 3.0) in *S.* Typhimurium after exposure to a > 100 mM SCFA mixture (Kwon et al., 2000), however, the concentration of SCFA used by Kwon et al. (2000) exceeded the concentration reported in our study. Similarly, *C. sakazakii* strains with high catalase activity and low *rpoS* expression can use acetate as a growth substrate in a nutrient-deprived environment (Álvarez-Ordóñez et al., 2012). The robust metabolism of *C. sakazakii*, in addition to the complex gut microbiota and metabolome, could indicate why, despite previous studies demonstrating the potent antimicrobial properties of acetate, there was a weak correlation between acetate concentration and *C. sakazakii* levels.

As with the other main SCFAs, propionate and butyrate have been reported to inhibit the growth of foodborne pathogens, despite these metabolites not being significantly correlated with the inhibition of *C. sakazakii* in this study. Butyrate has been noted to have the most consistent antimicrobial activity among the SCFAs, which may be attributed to stimulating the immune compartment and decreasing the expression of virulence genes in pathogens (Harrison et al., 2013). Additionally, propionate was also found to be better than acetate at inhibiting the growth of *C. sakazakii* in rehydrated infant formula, although it is not an acceptable additive in powdered infant formula (Oshima et al., 2012). The minimum inhibitory concentration (MIC) of butyrate and propionate against *S. enterica* strains in culture media was found to be approximately 3,750 μg/mL or around 45 mM on average (Lamas et al., 2019), but these concentrations exceed the SCFA levels generated in the infant SHIME. Both breast-fed and formula-fed infants lack the predominant butyrate-producing bacteria, such as *Eubacterium rectale* and *Faecalibacterium prausnitzii*, until approximately 6 months of age (Appert et al., 2020; Nilsen et al., 2020). Although the concentration of propionate and butyrate in the SHIME may not directly contribute to the inhibition of *C. sakazakii*, the combination of SCFAs in the colon environment may have a synergistic effect in inhibiting the growth of foodborne pathogens (Peng and Biswas, 2017). On the other hand, SCFA at sub-lethal concentrations may also be used by foodborne pathogens as a carbon source, thereby stimulating their growth (Dittoe et al., 2018). Although it is recognized that SCFAs have some role in inhibiting the growth of foodborne pathogens, it is important to consider that their efficacy depends on various factors including environmental pH, concentration and other bacterial strains and metabolites.

The infant SHIME microbiota was dominated by *Veillonella* and *Bacteroides*, which is in contrast to the commonly reported genera of a breast-fed infant which includes *Bifidobacterium*, *Staphylococcus*, *Streptococcus*, and *Lactobacillus* (Bäckhed et al., 2015; Moore and Townsend, 2019; Turroni et al., 2020; Ding et al., 2021). Although the composition of the infant gut microbiota can vary depending on various factors including diet, environment and delivery method, in this study, the SHIME microbiota composition may have been influenced by the basal feed used in the SHIME compartment. *Bacteroides*, *Veillonella*, and *Lachnoclostridium*, which represent some of the most abundant genera in the SHIME compartments from our study, can degrade mucin, a main component of the infant SHIME feed, to produce SCFAs (Flynn et al., 2016; Venegas et al., 2019; Raimondi et al., 2021). The ability of *Veillonella* to degrade mucin may also be a reason for its high abundance (>75% of the community abundance) in all SHIME compartments, which indicates that *Veillonella* can potentially outcompete other microorganisms within the SHIME compartments.

Alpha and beta-diversity indices were used to assess the stability of the SHIME microbiota once the *C. sakazakii* cocktail, with and without the treatments, had been introduced. The results showed that the first addition of the LAB + VGOS slightly increased the species diversity of SHIME 3 compared to the control and LAB, which may be a factor in the decline of *C. sakazakii* in both treatment periods. The gut microbiota can compete with pathogenic bacteria for nutrients, thereby limiting growth (Kamada et al., 2013; Tomar et al., 2015), and it is possible that a more diverse environment can contain other bacteria which use the same nutrients as *C. sakazakii*. The production of metabolites may be a result of a higher species diversity after the LAB + VGOS treatment, as the VGOS may be an additional nutrient source that can be metabolized into antimicrobial metabolites (Martin et al., 2019). In fact, the reduction in levels of *C. sakazakii* in the control and LAB + VGOS treatments may be a result of multiple factors involving the SHIME microbiota and metabolites.

To the best of the author’s knowledge, the impact of *C. sakazakii* on the infant gut microbiota has not been documented. In this study, the results suggest that the presence of *C. sakazakii* at 6 log CFU/mL, a concentration much higher than found in contaminated foods and in the environment, did not significantly affect the diversity of the infant SHIME microbiota. While some studies have shown that the presence of a foodborne pathogen can alter the gut microbiota in animal and *in vitro* gut models (Sannasiddappa et al., 2011; Iljazovic et al., 2021), others have highlighted the robustness of the human gut microbiota. Zhang et al. (2016) described a “like-to-like rule” for the human gut microbiota, which suggests that the native gut microbiota resists change to extraneous bacteria, such as foodborne pathogens and beneficial bacteria, that are ingested. The authors noted that a gut microbiota with a high abundance of native Enterobacteriaceae, which can include opportunistic pathogens like *Salmonella* and *Cronobacter* spp., can provide more favorable conditions for foodborne pathogens to grow. Conversely, another study by Stecher et al. (2010) found that *Lactobacillus reuteri* more effectively colonized a mouse microbiota containing a high abundance of lactobacilli compared to the ones with a lower abundance.

Despite previous studies reporting the efficacy of LAB to inhibit the growth of foodborne pathogens or the ability of probiotics to modulate the gut microbiota to exert a protective effect on the host (Sivieri et al., 2021), this study demonstrated the opposite. Species diversity and community abundance were similar between the LAB treatment and the control. Additionally, the metabolomic profiles of the SHIME suggest that the LAB treatment did not affect the SHIME metabolome. This may be due to the robustness of the microbiota as previously mentioned, where the lack of *Lactobacillus* and *Pediococcus* present in the SHIME prior to the treatment period resulted in poor colonization and/or adherence of the LAB within the vessels. However, the frequency of the LAB treatment should also be considered. Ren et al. (2018) found that the addition of a mixed LAB culture can not only prevent *Staphylococcus aureus* infection, but also maintain the gut microbiota in a mouse model. A key difference that may have minimized the efficacy of the LAB used in this study is the dosage or frequency of the LAB treatment. The study by Ren et al. (2018) used multiple feedings of the LAB throughout their experiment in an animal model, whereas in this study, the LAB was only introduced once in an *in vitro* gut model. The one-time inoculation of LAB may not have been sufficient to exhibit inhibitory effects on *C. sakazakii*, which would support the notion that probiotics should be ingested in adequate amounts to exert a health benefit (Hill et al., 2014).

Despite the controlled environment of the SHIME compartment, the results suggest that there is a vast network of interactions between and within the gut microbiota and metabolome. In this study, the associations between select metabolites and microbial genera were evaluated using a Spearman’s rank correlation and visualized on a heat map to provide insight into the reasons by which *C. sakazakii* levels decreased with and without any intervention. While the correlation matrix does not explicitly demonstrate the metabolic pathways within the infant SHIME, it does provide a statistical representation of several major interactions within the compartment based on the available data.

The correlation matrix of the control SHIME revealed a strong negative correlation between *Cronobacter* and *Bifidobacterium* spp., which is interesting as *Bifidobacterium* has been previously reported to inhibit the growth of enteropathogens (Servin, 2004; Vazquez-Gutierrez et al., 2016; Chichlowski et al., 2020). Infants that are premature or of low-birthweight may be more susceptible to *C. sakazakii* infection, as they may be missing some beneficial gut microbiota including *Bacteroides* and *Bifidobacterium* (Bäckhed et al., 2015; Yang et al., 2016; Cukrowska et al., 2020). Therefore, the findings of this study suggest that the presence of *Bifidobacterium* spp. could play a role in controlling *C. sakazakii* in a healthy infant gut. A possible mechanism of inhibition involves the production of organic acids, such as acetate, lactate and formate, which have a strong antagonistic effect against Gram-negative bacteria (Makras and De Vuyst, 2006). *Bifidobacterium* and other commensal gut microbiota can compete for nutrients with foodborne pathogens (Kamada et al., 2013; O’Callaghan and van Sinderen, 2016), which would further limit the ability for *C. sakazakii* to grow within the SHIME compartment. Despite the relatively low abundance of some gut microbiota, including *Bifidobacterium*, our results provide further evidence that the gut microbiota may play a significant biological role through synergistic interactions with other microorganisms (Claussen et al., 2017; Benjamino et al., 2018; Cena et al., 2021).

The Spearman’s rank correlation of the treatments revealed a stronger metabolite-metabolite relationship in the LAB + VGOS treatment as compared to the LAB treatment. The correlation matrix of the LAB + VGOS showed more defined metabolite-metabolite clusters compared to the LAB only treatment, and the significant associations between some metabolites such as propionate-acetate and propionate-formate. These patterns of correlations between metabolites and significant interactions were less evident within the LAB only treatment. These results provide further evidence that the reduction of *C. sakazakii* as a result of the LAB + VGOS treatment may be metabolite-dependent, since the regression analysis of the profiled metabolites showed a significant, but weak, correlation between acetate and *C. sakazakii*. The most abundant bacteria in the SHIME, namely *Bacteroides* and *Veillonella*, can produce acetate and other metabolites by metabolizing VGOS through similar metabolic pathways (Oliphant and Allen-Vercoe, 2019). It is important to note, however, that other SHIME microbiota could play a role in the complex metabolome-microbiota network by creating intermediary metabolites, e.g., formate, succinate and lactate, which are later converted into SCFAs (Oliphant and Allen-Vercoe, 2019; Tang et al., 2019). For example, Hinton and Hume (1995) found that a synergistic interaction between *Veillonella*, lactate and succinate could inhibit the growth of *S.* Typhimurium and *Salmonella* Enteritidis through the production of volatile fatty acids and a reduction of pH in broth media. Other studies on the effects of prebiotics on the gut metabolome and microbiota typically include consistent consumption of the prebiotic by the participants lasting multiple weeks, however, this SHIME experiment lasted 2 weeks, separated by 1-week intervals, with only one addition of the VGOS. In such cases, Tang et al. (2019) found that short-term diets had a stronger effect on the gut metabolome, as recently consumed nutrients are quickly metabolized by the gut microbiota without influencing the microbial composition.

The infant SHIME focused on the luminal portion of the colon, but the mucosal environment of the colon may be able to better simulate the colonization of pathogens, probiotics and the commensal gut bacteria (Van den Abbeele et al., 2012). Furthermore, the SHIME does not have an absorption unit, which allows SCFA to accumulate in the distal colon and therefore may not accurately reflect *in vivo* concentrations, immune compartment and maternal antibodies (Van de Wiele et al., 2015). Physical constraints also exist when one uses the triple SHIME. For example, the control used in this study was inoculated with only *C. sakazakii* in the first treatment period and sterile MRS media in the second treatment period. In order to evaluate the natural changes in community composition within the infant SHIME, it would have been interesting to conduct a SHIME trial that was not inoculated with *C. sakazakii* or the treatments. Additionally, as the start of the second treatment period occurred only a week after the start of the first treatment period, there may not have been sufficient time for the gut microbiota and metabolome to stabilize (Van de Wiele et al., 2015). The effects of the first treatment period on the gut microbiota and metabolome could have lingered and subsequently affected the efficacy of the second treatments. Overall, this continuous SHIME experiment gives insight into the short-term effects of different treatment combinations on the survival of *C. sakazakii* in the infant gut.

The focus of this study was on the inhibitory properties of a potential probiotic and synbiotic treatment, however, probiotics and synbiotics can exert antagonistic effects on foodborne pathogens in other ways. Additionally, probiotics and synbiotics can have beneficial effects on the intestinal epithelial cells by mitigating the adhesion of pathogens and promoting tight junction integrity (Collado et al., 2008; Piqué et al., 2019). Several factors, including the timing and dosage of probiotics and synbiotics should be considered when evaluating their beneficial effects on the host. The inconsistencies in methodology and reporting of the health benefits of probiotics, prebiotics and synbiotics, in addition to the wide selections of probiotics available, including *Bifidobacterium*, are a challenge in determining their optimal applications in infants (Van den Nieuwboer et al., 2014). The treatments used in this study were also directly applied to the colon in the absence of a food matrix such as PIF. Future experiments should also investigate the ability of probiotics and synbiotics to inhibit foodborne pathogens within a food matrix, such as PIF, the infant stomach/small intestine conditions, and a more thorough assessment on whether these LAB and LAB + VGOS treatments can be classified as probiotics or a synbiotic, respectively, and are safe to be added to PIF. Additionally, to better determine any symbiotic effects between the prebiotics and the LAB or probiotics, it may be worthwhile to test for the effects of only a prebiotic substrate on the inhibition of *C. sakazakii* as compared to the substrate in addition to LAB or other probiotics. The SHIME used in this study simulated the colonic conditions of a healthy infant, however, it would be beneficial for future studies to try and evaluate the efficacy of treatments in an *in vitro* model simulating the colonic conditions of low birth-weight and/or premature infants, as they are more vulnerable to infection by *C. sakazakii.*

To the best of our knowledge, this study is the first to evaluate the survival of *C. sakazakii* by using an *in vitro* gut model to mimic an infant’s colonic conditions. Altogether, our results suggest that *C. sakazakii* can be inhibited in the colon of healthy infants through competition for nutrients, production of antimicrobial compounds or a combination of mechanisms. In a healthy infant, it is possible that the presence of *C. sakazakii* is self-limiting due to the native gut microbiota. However, ingesting a treatment, such as synbiotics, could reduce the levels of *C. sakazakii* due to the production of metabolites such as SCFAs or through interactions between metabolites and the native gut microbiota. Furthermore, we provide evidence regarding the robustness of a healthy infant gut microbiota, albeit within a controlled environment simulating the colonic conditions of an infant, and for the potential biological significance of microbial genera present in relatively low abundance in the gut. Overall, our study suggests that the use of a synbiotic could be helpful in controlling potential *C. sakazakii* infection in infants.

## Data Availability Statement

The datasets presented in this study can be found in online repositories. The names of the repository/repositories and accession number(s) can be found in the article/Supplementary Material.

## Ethics statement

The studies involving human participants were reviewed and approved by the University of Guelph, Research Ethics Board (REB). Application to Involve Human Participants in Research. Written informed consent to participate in this study was provided by the participants’ legal guardian/next of kin.

## Author contributions

AK planned, conducted, and analyzed the experiments and the associated data, in addition to writing and revising the manuscript. VP, JF, and LG planned the experiment and revised the manuscript. All authors contributed to the article and approved the submitted version.

## Conflict of Interest

The authors declare that the research was conducted in the absence of any commercial or financial relationships that could be construed as a potential conflict of interest.

## Publisher’s Note

All claims expressed in this article are solely those of the authors and do not necessarily represent those of their affiliated organizations, or those of the publisher, the editors and the reviewers. Any product that may be evaluated in this article, or claim that may be made by its manufacturer, is not guaranteed or endorsed by the publisher.

## References

[B1] Abdulkadir B. Nelson A. Skeath T. Marrs E. C. Perry J. D. Cummings S. P. (2016). Routine use of probiotics in preterm infants: longitudinal impact on the microbiome and metabolome. *Neonatology* 109 239–247. 10.1159/000442936 26859305

[B2] Ackerberg T. S. Labuschagne L. I Lombard M. J. (2012). The use of prebiotics and probiotics in infant formula. *S. Afr. Fam. Pract.* 54 321–323. 10.1080/20786204.2012.10874243

[B3] Álvarez-Ordóñez A. Begley M. Hill C. (2012). Polymorphisms in *rpoS* and stress tolerance heterogeneity in natural isolates of *Cronobacter sakazakii*. *Appl. Environ. Microbiol.* 78 3975–3984. 10.1128/AEM.07835-11 22447602 PMC3346413

[B4] Appert O. Garcia A. R. Frei R. Roduit C. Constancias F. Neuzil-Bunesova V. (2020). Initial butyrate producers during infant gut microbiota development are endospore formers. *Environ. Microbiol.* 22 3909–3921. 10.1111/1462-2920.15167 32686173

[B5] Arnold C. N. McElhanon J. Lee A. Leonhart R. Siegele D. A. (2001). Global analysis of *Escherichia coli* gene expression during the acetate-induced acid tolerance response. *J. Bacteriol.* 183 2178–2186. 11244055 10.1128/JB.183.7.2178-2186.2001 PMC95122

[B6] Bäckhed F. Roswall J. Peng Y. Feng Q. Jia H. Kovatcheva-Datchary P. (2015). Dynamics and stabilization of the human gut microbiome during the first year of life. *Cell Host Microbe* 17 690–703. 10.1016/j.chom.2015.04.004 25974306

[B7] Bendjeddou K. Fons M. Strocker P. Sadoun D. (2012). Characterization and purification of a bacteriocin from *Lactobacillus paracasei* subsp. *paracasei* BMK2005, an intestinal isolate active against multidrug-resistant pathogens. *World J. Microbiol. Biotechnol.* 28 1543–1552. 10.1007/s11274-011-0958-1 22805936

[B8] Benjamino J. Lincoln S. Srivastava R. Graf J. (2018). Low-abundant bacteria drive compositional changes in the gut microbiota after dietary alteration. *Microbiome* 6 1–13. 10.1186/s40168-018-0469-5 29747692 PMC5944116

[B9] Bokulich N. A. Kaehler B. D. Rideout J. R. Dillon M. Bolyen E. Knight R. (2018). Optimizing taxonomic classification of marker gene amplicon sequences. *Microbiome* 6:90. 10.1186/s40168-018-0470-z 29773078 PMC5956843

[B10] Brink L. R. Mercer K. E. Piccolo B. D. Chintapalli S. V. Elolimy A. Bowlin A. K. (2020). Neonatal diet alters fecal microbiota and metabolome profiles at different ages in infants fed breast milk or formula. *Am. J. Clin. Nutr.* 111 1190–1202. 10.1093/ajcn/nqaa076 32330237 PMC7266684

[B11] Callahan B. J. McMurdie P. J. Rosen M. J. Han A. W. Johnson A. J. A. Holmes S. P. (2016). DADA2: high-resolution sample inference from Illumina amplicon data. *Nat. Methods* 13 581–583. 10.1038/nmeth.3869 27214047 PMC4927377

[B12] Caporaso J. G. Lauber C. L. Walters W. A. Berg-Lyons D. Lozupone C. A. Turnbaugh P. J. (2011). Global patterns of 16S rRNA diversity at a depth of millions of sequences per sample. *Proc. Nat. Acad. Sci. U.S.A.* 108 4516–4522. 10.1073/pnas.1000080107 20534432 PMC3063599

[B13] Cena J. Zhang J. Deng D. Damé-Teixeira N. Do T. (2021). Low-abundant microorganisms: the human microbiome’s dark matter, a scoping review. *Front. Cell. Infect. Microbiol.* 11:689197. 10.3389/fcimb.2021.689197 34136418 PMC8201079

[B14] Centers for Disease Control and Prevention [CDC] (2020a). *Frequently Asked Questions.* Atlanta: Centers for Disease Control and Prevention.

[B15] Centers for Disease Control and Prevention [CDC] (2020b). *Cronobacter Infection and Infants.* Atlanta: Centers for Disease Control and Prevention.

[B16] Centers for Disease Control and Prevention [CDC] (2022). *Cronobacter and Powdered Infant* Formula Investigation. Atlanta: Centers for Disease Control and Prevention.

[B17] Chichlowski M. Shah N. Wampler J. L. Wu S. S. Vanderhoof J. A. (2020). *Bifidobacterium longum* subspecies *infantis* (*B. infantis*) in pediatric nutrition: current state of knowledge. *Nutrients* 12:1581. 10.3390/nu12061581 32481558 PMC7352178

[B18] Chung H. J. Sim J. H. Min T. S. Choi H. K. (2018). Metabolomics and lipidomics approaches in the science of probiotics: a review. *J. Med. Food* 21 1086–1095. 10.1089/jmf.2017.4175 30004273

[B19] Claussen J. C. Skiecevičienė J. Wang J. Rausch P. Karlsen T. H. Lieb W. (2017). Boolean analysis reveals systematic interactions among low-abundance species in the human gut microbiome. *PLoS Comput. Biol.* 13:e1005361. 10.1371/journal.pcbi.1005361 28640804 PMC5480827

[B20] Collado M. C. Isolauri E. Salminen S. (2008). Specific probiotic strains and their combinations counteract adhesion of *Enterobacter sakazakii* to intestinal mucus. *FEMS Microbiol. Lett.* 285 58–64. 10.1111/j.1574-6968.2008.01211.x 18503543

[B21] Cukrowska B. Bierła J. B. Zakrzewska M. Klukowski M. Maciorkowska E. (2020). The relationship between the infant gut microbiota and allergy. The role of *Bifidobacterium breve* and prebiotic oligosaccharides in the activation of anti-allergic mechanisms in early life. *Nutrients* 12:946. 10.3390/nu12040946 32235348 PMC7230322

[B22] Dilli D. Aydin B. Fettah N. D. Özyazıcı E. Beken S. Zenciroğlu A. (2015). The ProPre-save study: effects of probiotics and prebiotics alone or combined on necrotizing enterocolitis in very low birth weight infants. *J. Pediatr.* 166 545–551. 10.1016/j.jpeds.2014.12.004 25596096

[B23] Ding M. Yang B. Khine W. W. T. Lee Y. K. Rahayu E. S. Ross R. P. (2021). The species-level composition of the fecal *Bifidobacterium* and *Lactobacillus* Genera in Indonesian children differs from that of their mothers. *Microorganisms* 9:1995. 10.3390/microorganisms9091995 PMC8467263 34576890

[B24] Dittoe D. K. Ricke S. C. Kiess A. S. (2018). Organic acids and potential for modifying the avian gastrointestinal tract and reducing pathogens and disease. *Front. Vet. Sci.* 5:216. 10.3389/fvets.2018.00216 30238011 PMC6136276

[B25] Fleming-Davies A. Jabbari S. Robertson S. L. Asih T. S. N. Lanzas C. Lenhart S. (2017). “Mathematical modeling of the effects of nutrient competition and bile acid metabolism by the gut microbiota on colonization resistance against *Clostridium difficile*,” in *Women in Mathematical Biology*, eds Layton A. Miller L. (Cham: Springer), 137–161. 10.1007/978-3-319-60304-9_8

[B26] Flynn J. M. Niccum D. Dunitz J. M. Hunter R. C. (2016). Evidence and role for bacterial mucin degradation in cystic fibrosis airway disease. *PLoS Pathog.* 12:e1005846. 10.1371/journal.ppat.1005846 27548479 PMC4993466

[B27] Fooks L. J. Gibson G. R. (2002). Probiotics as modulators of the gut flora. *Br. J. Nutr.* 88 S39–S49. 10.1079/BJN2002628 12215180

[B28] Gao Z. Daliri E. B. M. Wang J. U. N. Liu D. Chen S. Ye X. (2019). Inhibitory effect of lactic acid bacteria on foodborne pathogens: a review. *J. Food Prot.* 82 441–453. 10.4315/0362-028X.JFP-18-303 30794461

[B29] Gavriil A. Thanasoulia A. Skandamis P. N. (2020). Sublethal concentrations of undissociated acetic acid may not always stimulate acid resistance in *Salmonella enterica* sub. *enterica* serovar Enteritidis Phage Type 4: implications of challenge substrate associated factors. *PLoS One* 15:e0234999. 10.1371/journal.pone.0234999 32702039 PMC7377465

[B30] Ghini V. Tenori L. Pane M. Amoruso A. Marroncini G. Squarzanti D. F. (2020). Effects of Probiotics Administration on Human Metabolic Phenotype. *Metabolites* 10:396. 10.3390/metabo10100396 33036487 PMC7601401

[B31] Gillis C. C. Hughes E. R. Spiga L. Winter M. G. Zhu W. de Carvalho T. F. (2018). Dysbiosis-associated change in host metabolism generates lactate to support *Salmonella* growth. *Cell Host Microbe* 23 54–64. 10.1016/j.chom.2017.11.006 29276172 PMC5764812

[B32] Harrison L. M. Balan K. V. Babu U. S. (2013). Dietary fatty acids and immune response to food-borne bacterial infections. *Nutrients* 5 1801–1822. 10.3390/nu5051801 23698167 PMC3708349

[B33] Hill C. Guarner F. Reid G. Gibson G. R. Merenstein D. J. Pot B. (2014). Expert consensus document: the international scientific association for probiotics and prebiotics consensus statement on the scope and appropriate use of the term probiotic. *Nat. Rev. Gastroenterol. Hepatol.* 11 506–514. 10.1038/nrgastro.2014.66 24912386

[B34] Hinton A. Jr. Hume M. E. (1995). Synergism of lactate and succinate as metabolites utilized by *Veillonella* to inhibit the growth of *Salmonella* typhimurium and *Salmonella* enteritidis *in vitro*. *Avian Dis.* 39 309–316. 10.2307/1591872 7677651

[B35] Iljazovic A. Roy U. Gálvez E. J. Lesker T. R. Zhao B. Gronow A. (2021). Perturbation of the gut microbiome by *Prevotella* spp. enhances host susceptibility to mucosal inflammation. *Mucosal Immunol.* 14 113–124. 10.1038/s41385-020-0296-4 32433514 PMC7790746

[B36] Kalyantanda G. Shumyak L. Archibald L. K. (2015). Cronobacter species contamination of powdered infant formula and the implications for neonatal health. *Front. Pediatr.* 3:56. 10.3389/fped.2015.00056 26191519 PMC4489094

[B37] Kamada N. Chen G. Y. Inohara N. Núñez G. (2013). Control of pathogens and pathobionts by the gut microbiota. *Nat. Immunol.* 14 685–690. 10.1038/ni.2608 23778796 PMC4083503

[B38] Kassambara A. (2017). *Practical Guide to Principal Component Methods in R: PCA, M (CA), FAMD, MFA, HCPC, Factoextra*, Vol. 2. STHDA. Available online at: http://www.sthda.com/english/wiki/practical-guide-to-principal-component-methods-in-r

[B39] Ke A. Parreira V. Farber J. M. Goodridge L. (2022). Selection of a Potential Synbiotic against *Cronobacter sakazakii*. *J. Food Prot.* [Epub ahead of print]. 10.4315/JFP-22-048 35435968

[B40] Kent R. M. Fitzgerald G. F. Hill C. Stanton C. Paul Ross R. (2015). Novel approaches to improve the intrinsic microbiological safety of powdered infant milk formula. *Nutrients* 7 1217–1244. 10.3390/nu7021217 25685987 PMC4344585

[B41] Kirchberg F. F. Hellmuth C. Totzauer M. Uhl O. Closa-Monasterolo R. Escribano J. (2020). Impact of infant protein supply and other early life factors on plasma metabolome at 5.5 and 8 years of age: a randomized trial. *Int. J. Obes.* 44 69–81. 10.1038/s41366-019-0398-9 31300705 PMC7617055

[B42] Knaus U. G. Hertzberger R. Pircalabioru G. G. Yousefi S. P. M. Branco dos Santos F. (2017). Pathogen control at the intestinal mucosa–H_2_O_2_ to the rescue. *Gut Microbes* 8 67–74. 10.1080/19490976.2017.1279378 28080210 PMC5341913

[B43] Koh A. De Vadder F. Kovatcheva-Datchary P. Bäckhed F. (2016). From dietary fiber to host physiology: short-chain fatty acids as key bacterial metabolites. *Cell* 165 1332–1345. 10.1016/j.cell.2016.05.041 27259147

[B44] Kotsou M. G. Mitsou E. K. Oikonomou I. G. Kyriacou A. A. (2008). *In vitro* assessment of probiotic properties of *Lactobacillus* strains from infant gut microflora. *Food Biotechnol.* 22 1–17. 10.1080/08905430701707844

[B45] Kusmivati N. Wahyuningsih T. D. (2018). “Effect of Synbiotics *Lactobacillus casei* AP and Inulin Extract Dahlia pinnata L. in Enteropathogenic *Escherichia coli*-Induced Diarrhea,” in *2018 1st International Conference on Bioinformatics*. *Biotechnology, and Biomedical Engineering-Bioinformatics and Biomedical Engineering*, (Yogyakarta: IEEE), 1–6. 10.1109/BIOMIC.2018.8610642

[B46] Kwon Y. M. Park S. Y. Birkhold S. G. Ricke S. C. (2000). Induction of resistance of *Salmonella* Typhimurium to environmental stresses by exposure to short-chain fatty acids. *J. Food Sci.* 65 1037–1040. 10.1111/j.1365-2621.2000.tb09413.x

[B47] Lamas A. Regal P. Vázquez B. Cepeda A. Franco C. M. (2019). Short chain fatty acids commonly produced by gut microbiota influence *Salmonella enterica* motility, biofilm formation, and gene expression. *Antibiotics* 8:265. 10.3390/antibiotics8040265 31847278 PMC6963744

[B48] Lawhon S. D. Maurer R. Suyemoto M. Altier C. (2002). Intestinal short-chain fatty acids alter *Salmonella* typhimurium invasion gene expression and virulence through BarA/SirA. *Mol. Microbiol.* 46 1451–1464. 10.1046/j.1365-2958.2002.03268.x 12453229

[B49] Le Doare K. Holder B. Bassett A. Pannaraj P. S. (2018). Mother’s Milk: a purposeful contribution to the development of the infant microbiota and immunity. *Front. Immunol.* 9:361. 10.3389/fimmu.2018.00361 29599768 PMC5863526

[B50] LeBlanc J. G. Chain F. Martín R. Bermúdez-Humarán L. G. Courau S. Langella P. (2017). Beneficial effects on host energy metabolism of short-chain fatty acids and vitamins produced by commensal and probiotic bacteria. *Microb. Cell Fact.* 16 1–10. 10.1186/s12934-017-0691-z 28482838 PMC5423028

[B51] Likotrafiti E. Tuohy K. M. Gibson G. R. Rastall R. A. (2016). Antimicrobial activity of selected synbiotics targeted for the elderly against pathogenic *Escherichia coli* strains. *Int. J. Food Sci. Nutr.* 67 83–91. 10.3109/09637486.2015.1134444 26754553

[B52] Lund P. A. De Biase D. Liran O. Scheler O. Mira N. P. Cetecioglu Z. (2020). Understanding how microorganisms respond to acid pH is central to their control and successful exploitation. *Front. Microbiol.* 11:556140. 10.3389/fmicb.2020.556140 33117305 PMC7553086

[B53] Luoto R. Ruuskanen O. Waris M. Kalliomäki M. Salminen S. Isolauri E. (2014). Prebiotic and probiotic supplementation prevents rhinovirus infections in preterm infants: a randomized, placebo-controlled trial. *J. Allergy Clin. Immunol.* 133 405–413. 10.1016/j.jaci.2013.08.020 24131826 PMC7112326

[B54] Maifiah M. H. M. Creek D. J. Nation R. L. Forrest A. Tsuji B. T. Velkov T. (2017). Untargeted metabolomics analysis reveals key pathways responsible for the synergistic killing of colistin and doripenem combination against *Acinetobacter baumannii*. *Sci. Rep.* 7 1–12. 10.1038/srep45527 28358014 PMC5371981

[B55] Makras L. De Vuyst L. (2006). The *in vitro* inhibition of Gram-negative pathogenic bacteria by bifidobacteria is caused by the production of organic acids. *Int. Dairy J.* 16 1049–1057. 10.1016/j.idairyj.2005.09.006

[B56] Markowiak P. Śliżewska K. (2017). Effects of probiotics, prebiotics, and synbiotics on human health. *Nutrients* 9:1021. 10.3390/nu9091021 PMC5622781 28914794

[B57] Markowiak-Kopeć P. Śliżewska K. (2020). The effect of probiotics on the production of short-chain fatty acids by human intestinal microbiome. *Nutrients* 12:1107. 10.3390/nu12041107 32316181 PMC7230973

[B58] Martin A. M. Sun E. W. Rogers G. B. Keating D. J. (2019). The influence of the gut microbiome on host metabolism through the regulation of gut hormone release. *Front. Physiol.* 10:428. 10.3389/fphys.2019.00428 31057420 PMC6477058

[B59] Martin F. P. J. Sprenger N. Montoliu I. Rezzi S. Kochhar S. Nicholson J. K. (2010). Dietary modulation of gut functional ecology studied by fecal metabonomics. *J. Proteome Res.* 9 5284–5295. 10.1021/pr100554m 20806900

[B60] Martín R. Langa S. Reviriego C. Jimínez E. Marín M. L. Xaus J. (2003). Human milk is a source of lactic acid bacteria for the infant gut. *J. Pediatr.* 143 754–758. 10.1016/j.jpeds.2003.09.028 14657823

[B61] McMurdie P. J. Holmes S. (2013). phyloseq: an R package for reproducible interactive analysis and graphics of microbiome census data. *PLoS One* 8:e61217. 10.1371/journal.pone.0061217 23630581 PMC3632530

[B62] Moore R. E. Townsend S. D. (2019). Temporal development of the infant gut microbiome. *Open Biol.* 9:190128. 10.1098/rsob.190128 31506017 PMC6769289

[B63] Murphy J. Mahony J. Fitzgerald G. F. van Sinderen D. (2017). “Bacteriophages infecting lactic acid bacteria,” in *Cheese*, eds Fox M. P. C. P. P.F. Everett D. (Cambridge: Academic Press), 249–272. 10.1016/B978-0-12-417012-4.00010-7

[B64] Nandhini L. P. Biswal N. Adhisivam B. Mandal J. Bhat B. V. Mathai B. (2016). Synbiotics for decreasing incidence of necrotizing enterocolitis among preterm neonates–a randomized controlled trial. *J. Matern.-Fetal Neonatal Med.* 29 821–825. 10.3109/14767058.2015.1019854 25754214

[B65] Ng K. M. Ferreyra J. A. Higginbottom S. K. Lynch J. B. Kashyap P. C. Gopinath S. (2013). Microbiota-liberated host sugars facilitate post-antibiotic expansion of enteric pathogens. *Nature* 502 96–99. 10.1038/nature12503 23995682 PMC3825626

[B66] Nilsen M. Madelen Saunders C. Leena Angell I. Arntzen M. Ø Lødrup Carlsen K. C. Carlsen K. H. (2020). Butyrate levels in the transition from an infant-to an adult-like gut microbiota correlate with bacterial networks associated with *Eubacterium rectale* and *Ruminococcus gnavus*. *Genes* 11:1245. 10.3390/genes11111245 33105702 PMC7690385

[B67] O’Callaghan A. van Sinderen D. (2016). Bifidobacteria and their role as members of the human gut microbiota. *Front. Microbiol.* 7:925. 10.3389/fmicb.2016.00925 27379055 PMC4908950

[B68] O’Connell T. M. (2020). The application of metabolomics to probiotic and prebiotic interventions in human clinical studies. *Metabolites* 10:120. 10.3390/metabo10030120 32213886 PMC7143099

[B69] Oksanen J. Blanchet F. G. Friendly M. Kindt R. Legendre P. McGlinn D. (2020). *Vegan: Community Ecology Package.* Available Online at: https://CRAN.R-project.org/package=vegan (accessed November 6, 2021).

[B70] Oliphant K. Allen-Vercoe E. (2019). Macronutrient metabolism by the human gut microbiome: major fermentation by-products and their impact on host health. *Microbiome* 7 1–15. 10.1186/s40168-019-0704-8 31196177 PMC6567490

[B71] Oshima S. Rea M. C. Lothe S. Morgan S. Begley M. O’Connor P. M. (2012). Efficacy of organic acids, bacteriocins, and the lactoperoxidase system in inhibiting the growth of *Cronobacter* spp. in rehydrated infant formula. *J. Food Prot.* 75 1734–1742. 10.4315/0362-028X.JFP-12-066 23043820

[B72] Pandey K. R. Naik S. R. Vakil B. V. (2015). Probiotics, prebiotics and synbiotics - a review. *J. Food Sci. Technol.* 52 7577–7587. 10.1007/s13197-015-1921-1 26604335 PMC4648921

[B73] Papagianni M. Anastasiadou S. (2009). Pediocins: the bacteriocins of *Pediococci*. Sources, production, properties and applications. *Microb. Cell Fact.* 8 1–16. 10.1186/1475-2859-8-3 19133115 PMC2634753

[B74] Patnode M. L. Beller Z. W. Han N. D. Cheng J. Peters S. L. Terrapon N. (2019). Interspecies competition impacts targeted manipulation of human gut bacteria by fiber-derived glycans. *Cell* 179 59–73. 10.1016/j.cell.2019.08.011 31539500 PMC6760872

[B75] Pehlevan O. S. Benzer D. Gursoy T. Karatekin G. Ovali F. (2020). Synbiotics use for preventing sepsis and necrotizing enterocolitis in very low birth weight neonates: a randomized controlled trial. *Clin. Exp. Pediatr.* 63:226. 10.3345/cep.2019.00381 32023397 PMC7303425

[B76] Peng M. Biswas D. (2017). Short chain and polyunsaturated fatty acids in host gut health and foodborne bacterial pathogen inhibition. *Crit. Rev. Food Sci. Nutr.* 57 3987–4002. 10.1080/10408398.2016.1203286 27438132

[B77] Phan M. Momin S. R. Senn M. K. Wood A. C. (2019). Metabolomic insights into the effects of breast milk versus formula milk feeding in infants. *Curr. Nutr. Rep.* 8 295–306. 10.1007/s13668-019-00284-2 31203566

[B78] Piatek J. Krauss H. Ciechelska-Rybarczyk A. Bernatek M. Wojtyla-Buciora P. Sommermeyer H. (2020). In-Vitro Growth Inhibition of Bacterial Pathogens by Probiotics and a Synbiotic: Product Composition Matters. *Int. J. Environ. Res. Public Health* 17:3332. 10.3390/ijerph17093332 32403297 PMC7246756

[B79] Piqué N. Berlanga M. Miñana-Galbis D. (2019). Health benefits of heat-killed (Tyndallized) probiotics: an overview. *Int. J. Mol. Sci.* 20:2534. 10.3390/ijms20102534 31126033 PMC6566317

[B80] Prudêncio C. V. Dos Santos M. T. Vanetti M. C. D. (2015). Strategies for the use of bacteriocins in Gram-negative bacteria: relevance in food microbiology. *J. Food Sci. Technol.* 52 5408–5417. 10.1007/s13197-014-1666-2 26344957 PMC4554667

[B81] Quast C. Pruesse E. Yilmaz P. Gerken J. Schweer T. Yarza P. (2012). The SILVA ribosomal RNA gene database project: improved data processing and web-based tools. *Nucleic Acids Res.* 41 D590–D596. 10.1093/nar/gks1219 23193283 PMC3531112

[B82] Raimondi S. Musmeci E. Candeliere F. Amaretti A. Rossi M. (2021). Identification of mucin degraders of the human gut microbiota. *Sci. Rep.* 11 1–10. 10.1038/s41598-021-90553-4 34045537 PMC8159939

[B83] Ren D. Gong S. Shu J. Zhu J. Liu H. Chen P. (2018). Effects of mixed lactic acid bacteria on intestinal microbiota of mice infected with *Staphylococcus aureus*. *BMC Microbiol.* 18:109. 10.1186/s12866-018-1245-1 30189834 PMC6127954

[B84] Rodríguez-Rojas A. Kim J. J. Johnston P. R. Makarova O. Eravci M. Weise C. (2020). Non-lethal exposure to H2O2 boosts bacterial survival and evolvability against oxidative stress. *PLoS Genet.* 16:e1008649. 10.1371/journal.pgen.1008649 32163413 PMC7093028

[B85] Rolfe R. D. (2000). The role of probiotic cultures in the control of gastrointestinal health. *J. Nutr.* 130 396S–402S. 10.1093/jn/130.2.396S 10721914

[B86] Rubio R. Jofré A. Martín B. Aymerich T. Garriga M. (2014). Characterization of lactic acid bacteria isolated from infant faeces as potential probiotic starter cultures for fermented sausages. *Food Microbiol.* 38 303–311. 10.1016/j.fm.2013.07.015 24290655

[B87] Sakr E. A. Massoud M. I. (2021). Impact of prebiotic potential of stevia sweeteners-sugar used as synbiotic preparation on antimicrobial, antibiofilm, and antioxidant activities. *LWT* 144:111260. 10.1016/j.lwt.2021.111260

[B88] Sannasiddappa T. H. Costabile A. Gibson G. R. Clarke S. R. (2011). The influence of *Staphylococcus aureus* on gut microbial ecology in an *in vitro* continuous culture human colonic model system. *PLoS One* 6:e23227. 10.1371/journal.pone.0023227 21858036 PMC3153491

[B89] Servin A. L. (2004). Antagonistic activities of lactobacilli and bifidobacteria against microbial pathogens. *FEMS Microbiol. Rev.* 28 405–440. 10.1016/j.femsre.2004.01.003 15374659

[B90] Shanmugasundaram R. Mortada M. Cosby D. E. Singh M. Applegate T. J. Syed B. (2019). Synbiotic supplementation to decrease *Salmonella* colonization in the intestine and carcass contamination in broiler birds. *PLoS One* 14:e0223577. 10.1371/journal.pone.0223577 31600299 PMC6786831

[B91] Sieniawska E. Georgiev M. I. (2022). Metabolomics: towards acceleration of antibacterial plant-based leads discovery. *Phytochem. Rev.* 21, 765–781. 10.1007/s11101-021-09762-4

[B92] Simons A. Alhanout K. Duval R. E. (2020). Bacteriocins, antimicrobial peptides from bacterial origin: overview of their biology and their impact against multidrug-resistant bacteria. *Microorganisms* 8:639. 10.3390/microorganisms8050639 PMC7285073 32349409

[B93] Sivieri K. Bianchi F. Tallarico M. A. Rossi E. A. (2011). Fermentation by gut microbiota cultured in a simulator of the human intestinal microbial ecosystem is improved by probiotic *Enterococcus faecium* CRL 183. *Funct. Foods Health Dis.* 1 389–402. 10.31989/ffhd.v1i10.119

[B94] Sivieri K. Sáyago-Ayerdi S. G. Binetti A. G. (2021). Insights of Gut Microbiota: probiotics and Bioactive Compounds. *Front. Microbiol.* 12:780596. 10.3389/fmicb.2021.780596 34790188 PMC8591445

[B95] Solís G. de Los Reyes-Gavilan C. G. Fernández N. Margolles A. Gueimonde M. (2010). Establishment and development of lactic acid bacteria and bifidobacteria microbiota in breast-milk and the infant gut. *Anaerobe* 16 307–310. 10.1016/j.anaerobe.2010.02.004 20176122

[B96] Sreenivasa B. Kumar P. S. Suresh Babu M. T. Ragavendra K. (2015). Role of synbiotics in the prevention of necrotizing enterocolitis in preterm neonates: a randomized controlled trial. *Int. J. Contemp. Pediatr.* 2 127–130. 10.5455/2349-3291.ijcp20150512

[B97] Stecher B. Chaffron S. Käppeli R. Hapfelmeier S. Freedrich S. Weber T. C. (2010). Like will to like: abundances of closely related species can predict susceptibility to intestinal colonization by pathogenic and commensal bacteria. *PLoS Pathog.* 6:e1000711. 10.1371/journal.ppat.1000711 20062525 PMC2796170

[B98] Stewart C. J. Embleton N. D. Marrs E. C. Smith D. P. Fofanova T. Nelson A. (2017). Longitudinal development of the gut microbiome and metabolome in preterm neonates with late onset sepsis and healthy controls. *Microbiome* 5 1–11. 10.1186/s40168-017-0295-1 28701177 PMC5508794

[B99] Swanson K. S. Gibson G. R. Hutkins R. Reimer R. A. Reid G. Verbeke K. (2020). The International Scientific Association for Probiotics and Prebiotics (ISAPP) consensus statement on the definition and scope of synbiotics. *Nat. Rev. Gastroenterol. Hepatol.* 17 687–701. 10.1038/s41575-020-0344-2 32826966 PMC7581511

[B100] Tabashsum Z. Peng M. Bernhardt C. Patel P. Carrion M. Biswas D. (2019). Synbiotic-like effect of linoleic acid overproducing *Lactobacillus casei* with berry phenolic extracts against pathogenesis of enterohemorrhagic *Escherichia coli*. *Gut Pathog.* 11:41. 10.1186/s13099-019-0320-y 31372184 PMC6661093

[B101] Tang Z. Z. Chen G. Hong Q. Huang S. Smith H. M. Shah R. D. (2019). Multi-omic analysis of the microbiome and metabolome in healthy subjects reveals microbiome-dependent relationships between diet and metabolites. *Front. Genet.* 10:454. 10.3389/fgene.2019.00454 PMC6534069 31164901

[B102] Tharrington G. Sorrells K. M. (1992). Inhibition of *Listeria monocytogenes* by milk culture filtrates from *Lactobacillus delbrueckii* subsp. *lactis*. *J. Food Prot.* 55 542–544. 10.4315/0362-028x-55.7.542 31071902

[B103] Tian X. Yu Q. Yao D. Shao L. Liang Z. Jia F. (2018). New insights into the response of metabolome of *Escherichia coli* O157: H7 to ohmic heating. *Front. Microbiol.* 9:2936. 10.3389/fmicb.2018.02936 30574129 PMC6291463

[B104] Tomar S. K. Anand S. Sharma P. Sangwan V. Mandal S. (2015). “Role of probiotic, prebiotics, synbiotics and postbiotics in inhibition of pathogens,” in *The Battle against Microbial Pathogens: Basic Science, Technological Advances and Educational Programs*, ed. Méndez-Vilas A. (Badajoz: Formatex Research Centre), 717–732.

[B105] Turroni F. Milani C. Duranti S. Lugli G. A. Bernasconi S. Margolles A. (2020). The infant gut microbiome as a microbial organ influencing host well-being. *Ital. J. Pediatr.* 46 1–13. 10.1186/s13052-020-0781-0 32024556 PMC7003403

[B106] Van de Wiele T. Van den Abbeele P. Ossieur W. Possemiers S. Marzorati M. (2015). “The simulator of the human intestinal microbial ecosystem (SHIME^®^),” in *The Impact of Food Bioactives on Health*, eds Verhoeckx K. Cotter P. ópez-Expósito I. L. Kleiveland C. Lea T. Mackie A. (Cham: Springer), 305–317. 10.1007/978-3-319-16104-4_27 29787058

[B107] Van den Abbeele P. Grootaert C. Marzorati M. Possemiers S. Verstraete W. Gérard P. (2010). Microbial community development in a dynamic gut model is reproducible, colon region specific, and selective for Bacteroidetes and Clostridium cluster IX. *Appl. Environ. Microbiol.* 76 5237–5246. 10.1128/AEM.00759-10 20562281 PMC2916472

[B108] Van den Abbeele P. Roos S. Eeckhaut V. MacKenzie D. A. Derde M. Verstraete W. (2012). Incorporating a mucosal environment in a dynamic gut model results in a more representative colonization by lactobacilli. *Microb. Biotechnol.* 5 106–115. 10.1111/j.1751-7915.2011.00308.x 21989255 PMC3815277

[B109] Van den Nieuwboer M. Claassen E. Morelli L. Guarner F. Brummer R. J. (2014). Probiotic and synbiotic safety in infants under two years of age. *Benef. Microbes* 5 45–60. 10.3920/BM2013.0046 24463207

[B110] Vandenplas Y. Zakharova I. Dmitrieva Y. (2015). Oligosaccharides in infant formula: more evidence to validate the role of prebiotics. *Br. J. Nutr.* 113 1339–1344. 10.1017/S0007114515000823 25989994

[B111] Vazquez-Gutierrez P. de Wouters T. Werder J. Chassard C. Lacroix C. (2016). High iron-sequestrating bifidobacteria inhibit enteropathogen growth and adhesion to intestinal epithelial cells *in vitro*. *Front. Microbiol.* 7:1480. 10.3389/fmicb.2016.01480 27713730 PMC5031772

[B112] Venegas D. P. De La Fuente M. K. Landskron G. González M. J. Quera R. Dijkstra G. (2019). Short chain fatty acids (SCFAs) mediated gut epithelial and immune regulation and its relevance for inflammatory bowel diseases. *Front. Immunol.* 10:277. 10.3389/fimmu.2019.00277 30915065 PMC6421268

[B113] Vitali B. Ndagijimana M. Cruciani F. Carnevali P. Candela M. Guerzoni M. E. (2010). Impact of a synbiotic food on the gut microbial ecology and metabolic profiles. *BMC Microbiol.* 10:4. 10.1186/1471-2180-10-4 20055983 PMC2806344

[B114] Vongbhavit K. Underwood M. A. (2016). Prevention of Necrotizing Enterocolitis Through Manipulation of the Intestinal Microbiota of the Premature Infant. *Clin. Ther.* 38 716–732. 10.1016/j.clinthera.2016.01.006 26872618 PMC4902014

[B115] Wilson K. H. Perini F. (1988). Role of competition for nutrients in suppression of *Clostridium difficile* by the colonic microflora. *Infect. Immun.* 56 2610–2614. 10.1128/iai.56.10.2610-2614.1988 3417352 PMC259619

[B116] Yang I. Corwin E. J. Brennan P. A. Jordan S. Murphy J. R. Dunlop A. (2016). The infant microbiome: implications for infant health and neurocognitive development. *Nurs. Res.* 65:76. 10.1097/NNR.0000000000000133 PMC4681407 26657483

[B117] Zhang C. Derrien M. Levenez F. Brazeilles R. Ballal S. A. Kim J. (2016). Ecological robustness of the gut microbiota in response to ingestion of transient food-borne microbes. *ISME J.* 10 2235–2245. 10.1038/ismej.2016.13 26953599 PMC4989305

[B118] Zhang Y. Xie Y. Tang J. Wang S. Wang L. Zhu G. (2020). Thermal inactivation of *Cronobacter sakazakii* ATCC 29544 in powdered infant formula milk using thermostatic radio frequency. *Food Control* 114:107270. 10.1016/j.foodcont.2020.1070

