# Supplementary material for: Inhibition of Cronobacter sakazakii in an infant simulator of the human intestinal microbial ecosystem using a potential synbiotic
Source: Front Microbiol. 2022 Jul 15;13:947624. doi: 10.3389/fmicb.2022.947624 (PMC9335077; doi:10.3389/fmicb.2022.947624)
Supplement: Supplementary file 1 [file Table_1.DOCX]

Supplementary table 1. Selected bacterial strains used in the infant SHIME.

| **Strain** | **Original source** | **Description** |
| --- | --- | --- |
| *Lactobacillus plantarum* KG-14365 | Unknown | Farber Lab Culture Collection |
| *Lactobacillus plantarum* ATCC 202195 | Infant stool | Panigrahi et al., 2017; Wright et al., 2020; Pell et al., 2021 |
| *Lactobacillus rhamnosus* | Unknown | Farber’s Lab Culture Collection |
| *Pediococcus pentosaceus* E2 | Unknown | Farber’s Lab Culture Collection |
| *Pediococcus pentosaceus* E4 | Unknown | Farber’s Lab Culture Collection |
| *Pediococcus pentosaceus* E8 | Unknown | Farber’s Lab Culture Collection |
| *C. sakazakii* 2855 | Clinical (Sick Kids Hospital, 1981) | Health Canada |
| *C. sakazakii* 2871 | Food (infant formula; Mead Johnson Co.) | Health Canada |
| *C. sakazakii* 3199 | Environment (food processing; FDA) | Health Canada |
| *C. sakazakii* 3253 | Environment (hospital; The Netherlands) | Health Canada |
