# Supplementary figures and images for: Inhibition of Cronobacter sakazakii in an infant simulator of the human intestinal microbial ecosystem using a potential synbiotic
Source: Front Microbiol. 2022 Jul 15;13:947624. doi: 10.3389/fmicb.2022.947624 (PMC9335077; doi:10.3389/fmicb.2022.947624)

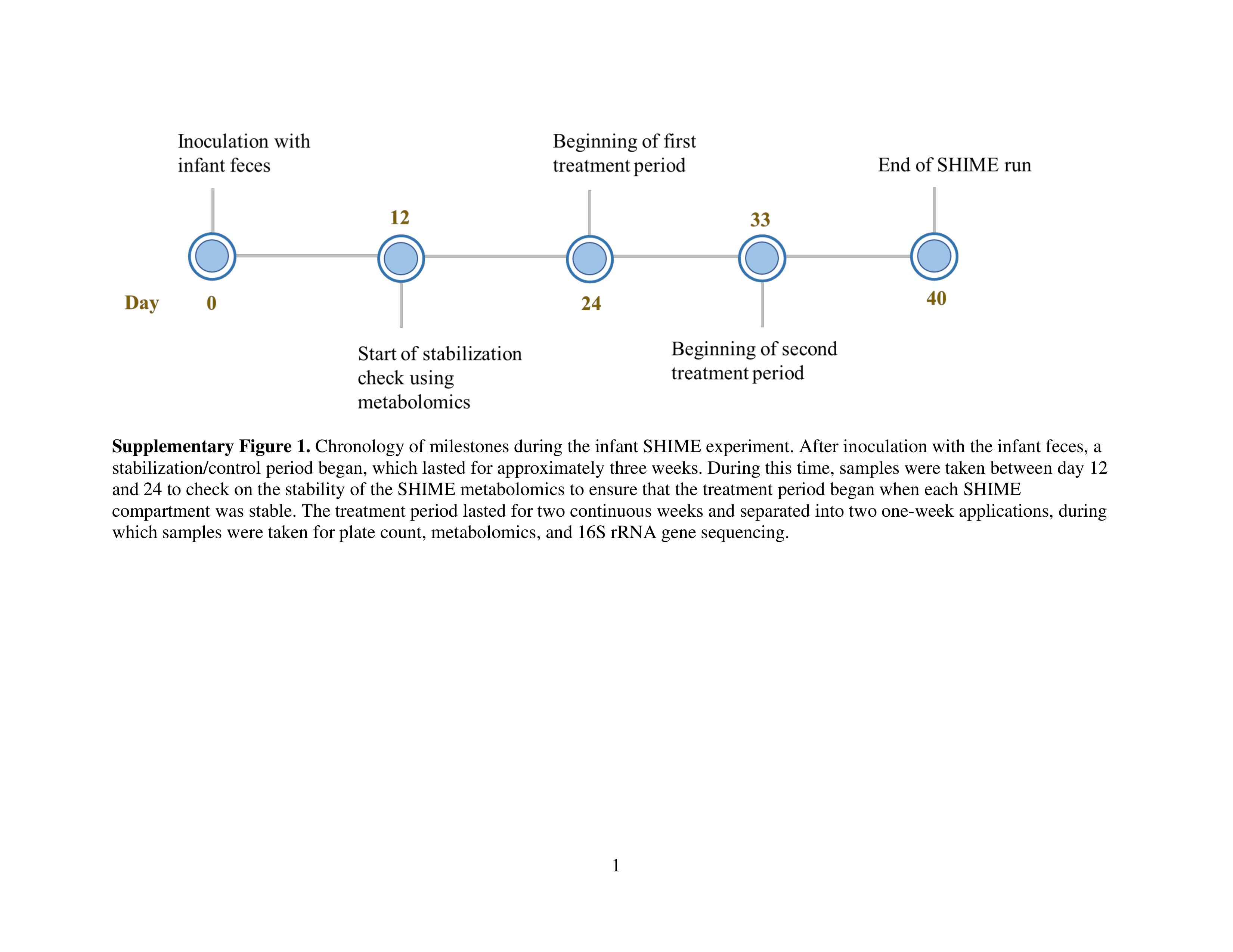

Supplement: Supplementary file 2 [file Image_1.JPEG]

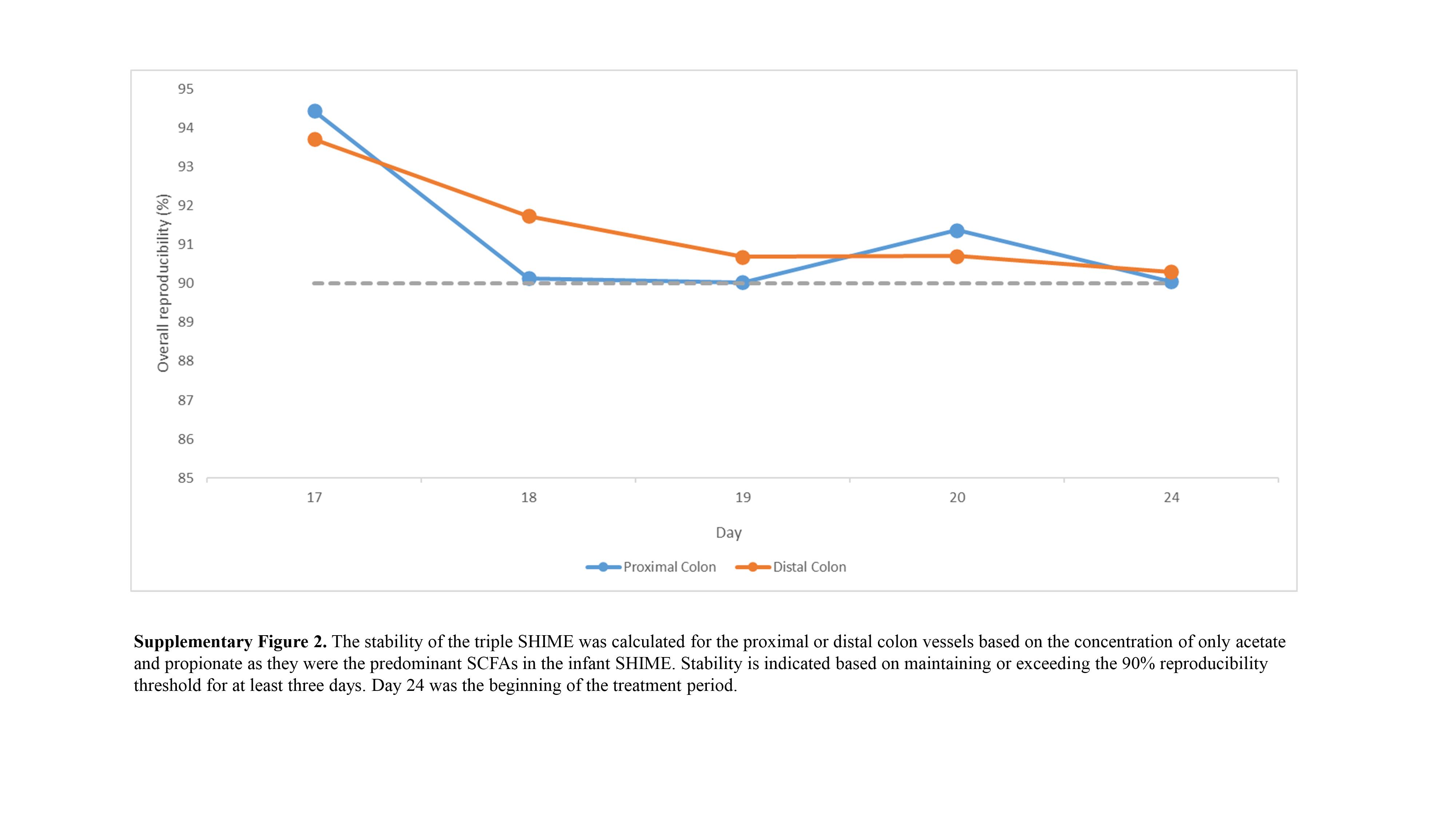

Supplement: Supplementary file 3 [file Image_2.JPEG]

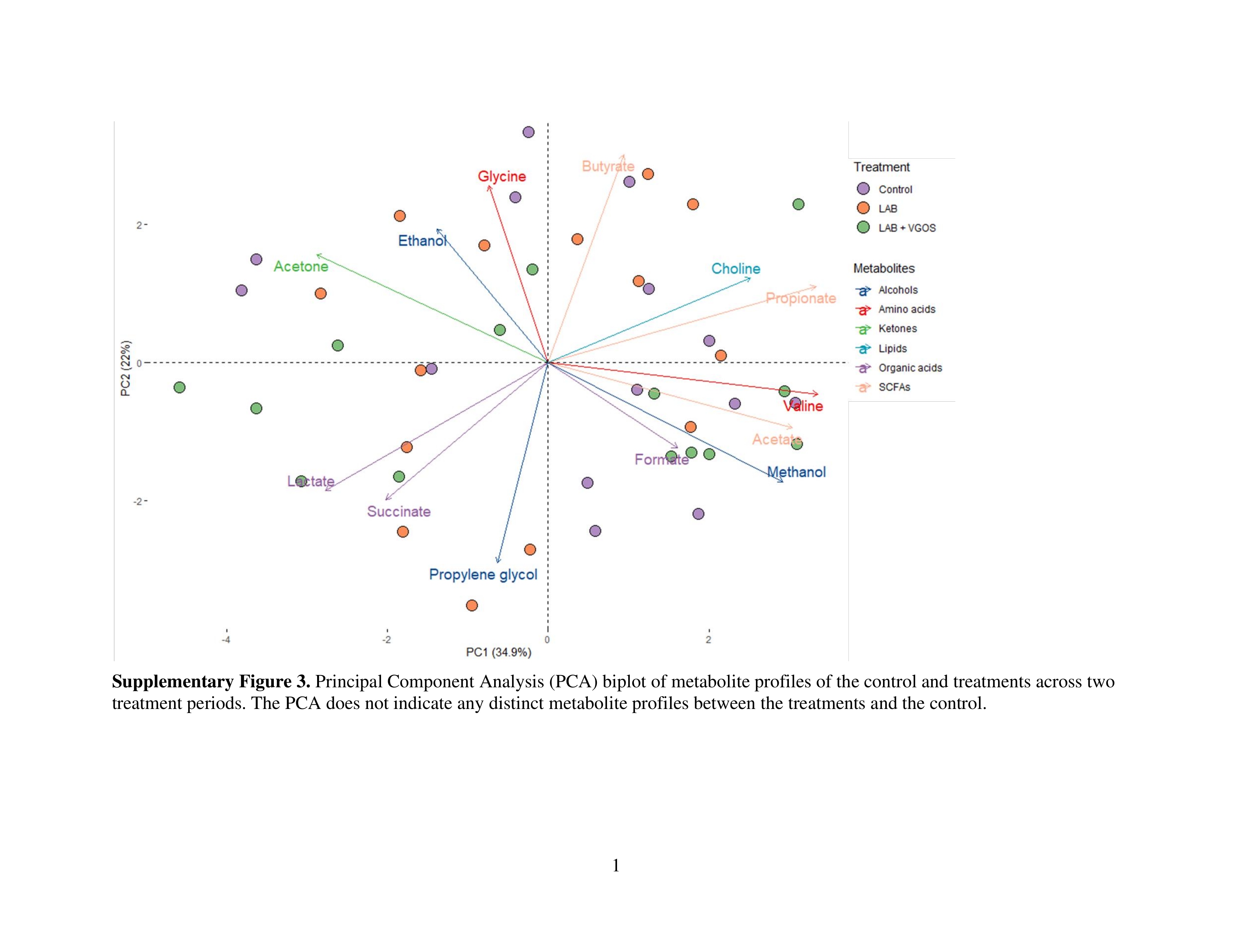

Supplement: Supplementary file 4 [file Image_3.JPEG]

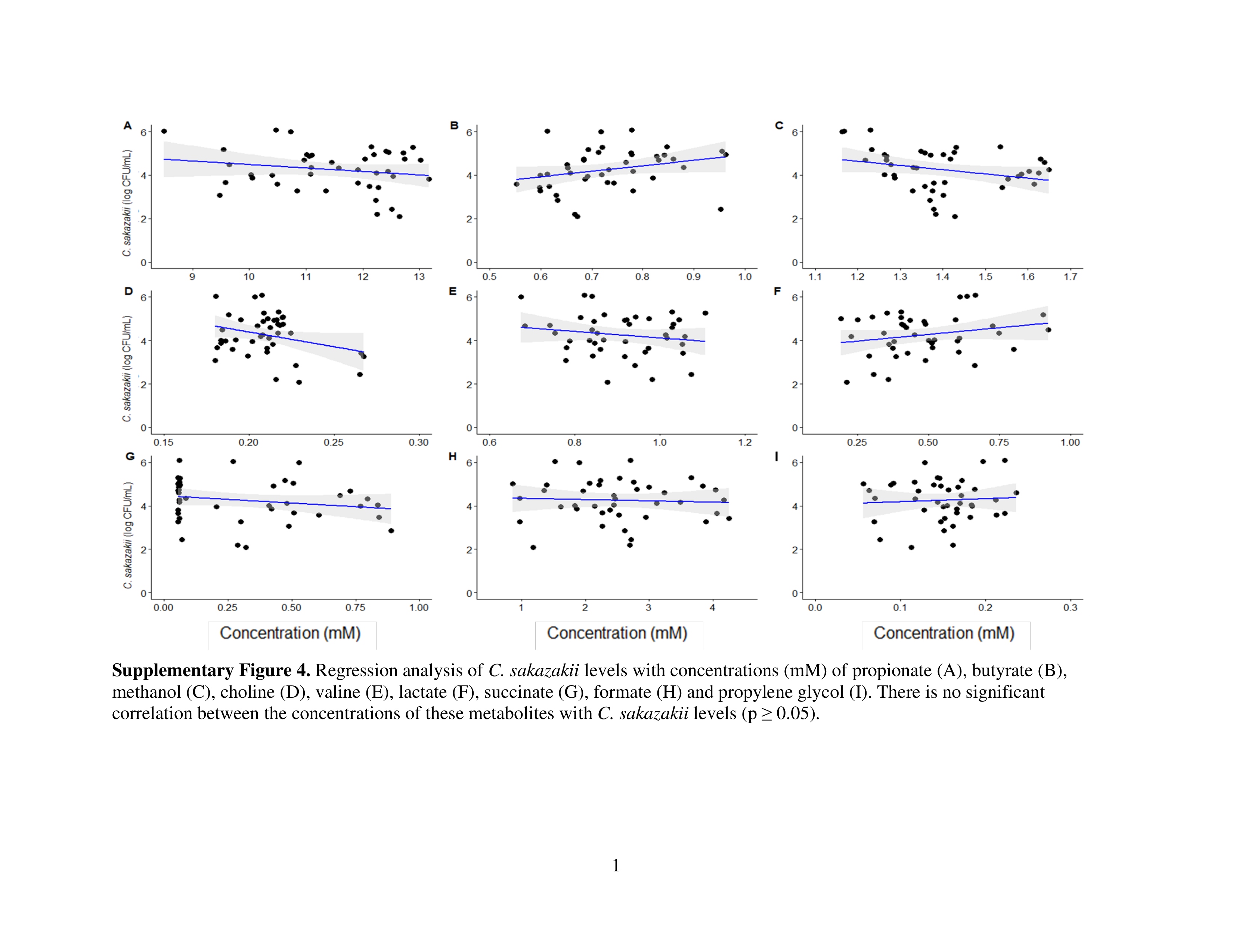

Supplement: Supplementary file 5 [file Image_4.JPEG]

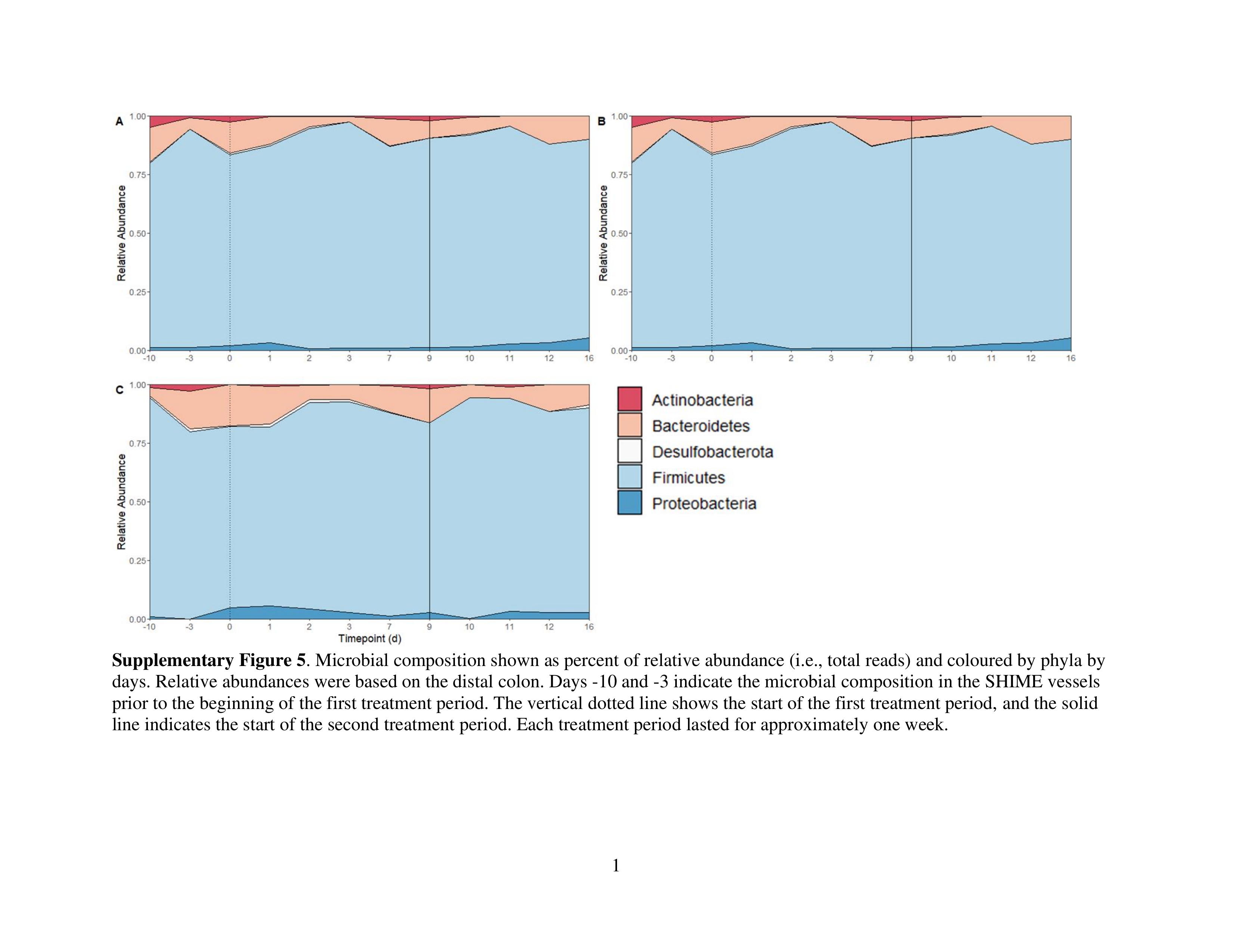

Supplement: Supplementary file 6 [file Image_5.JPEG]

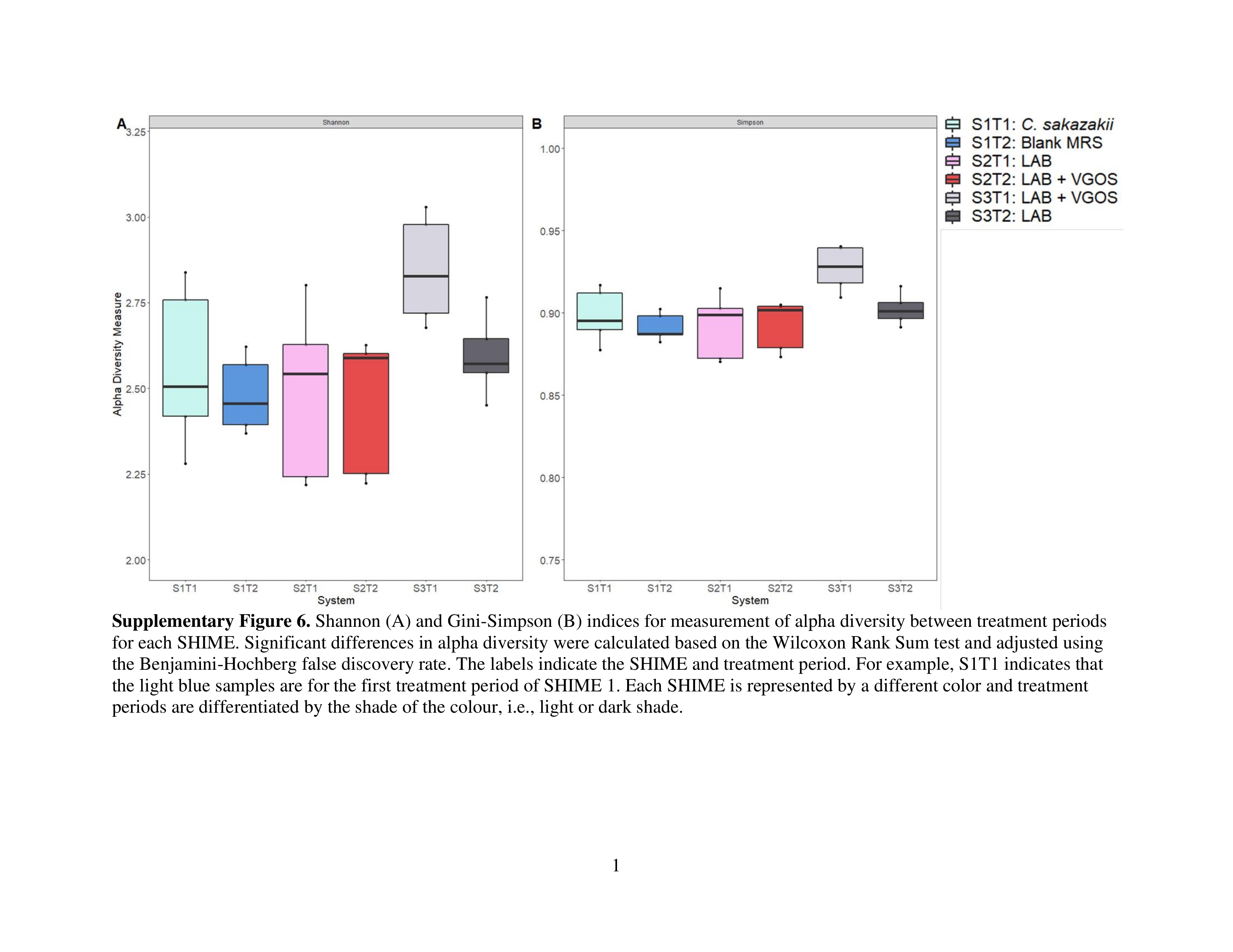

Supplement: Supplementary file 7 [file Image_6.JPEG]

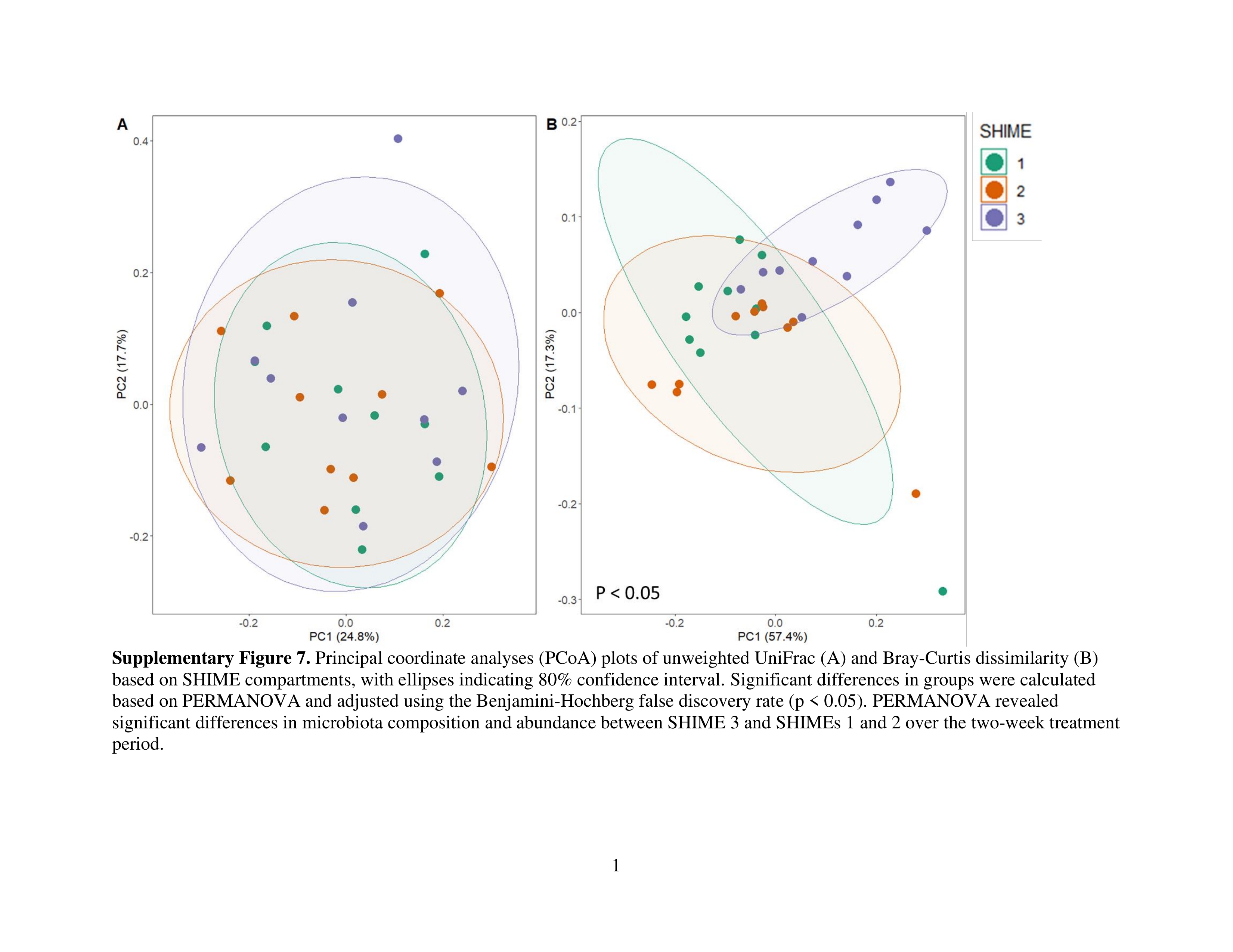

Supplement: Supplementary file 8 [file Image_7.JPEG]

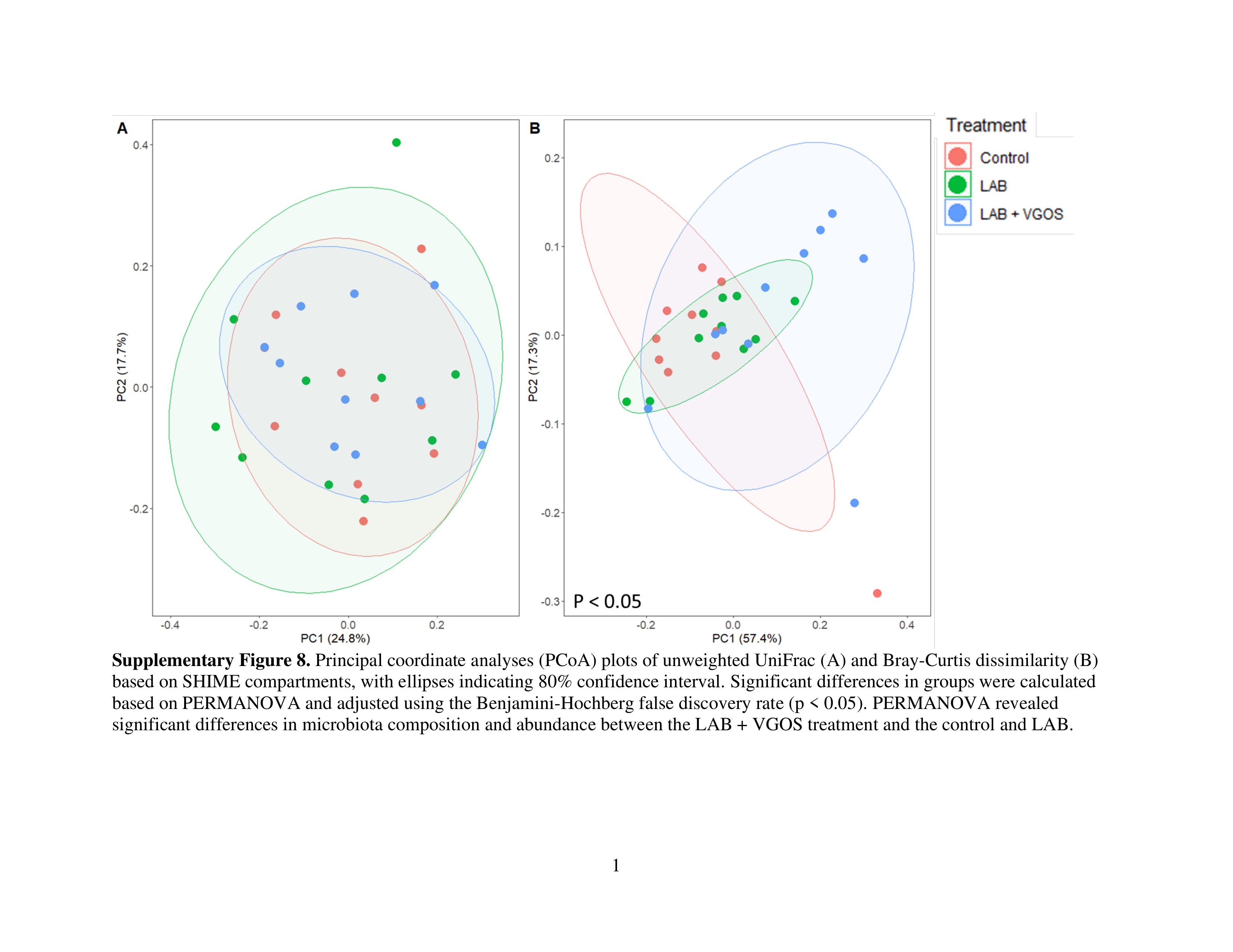

Supplement: Supplementary file 9 [file Image_8.JPEG]

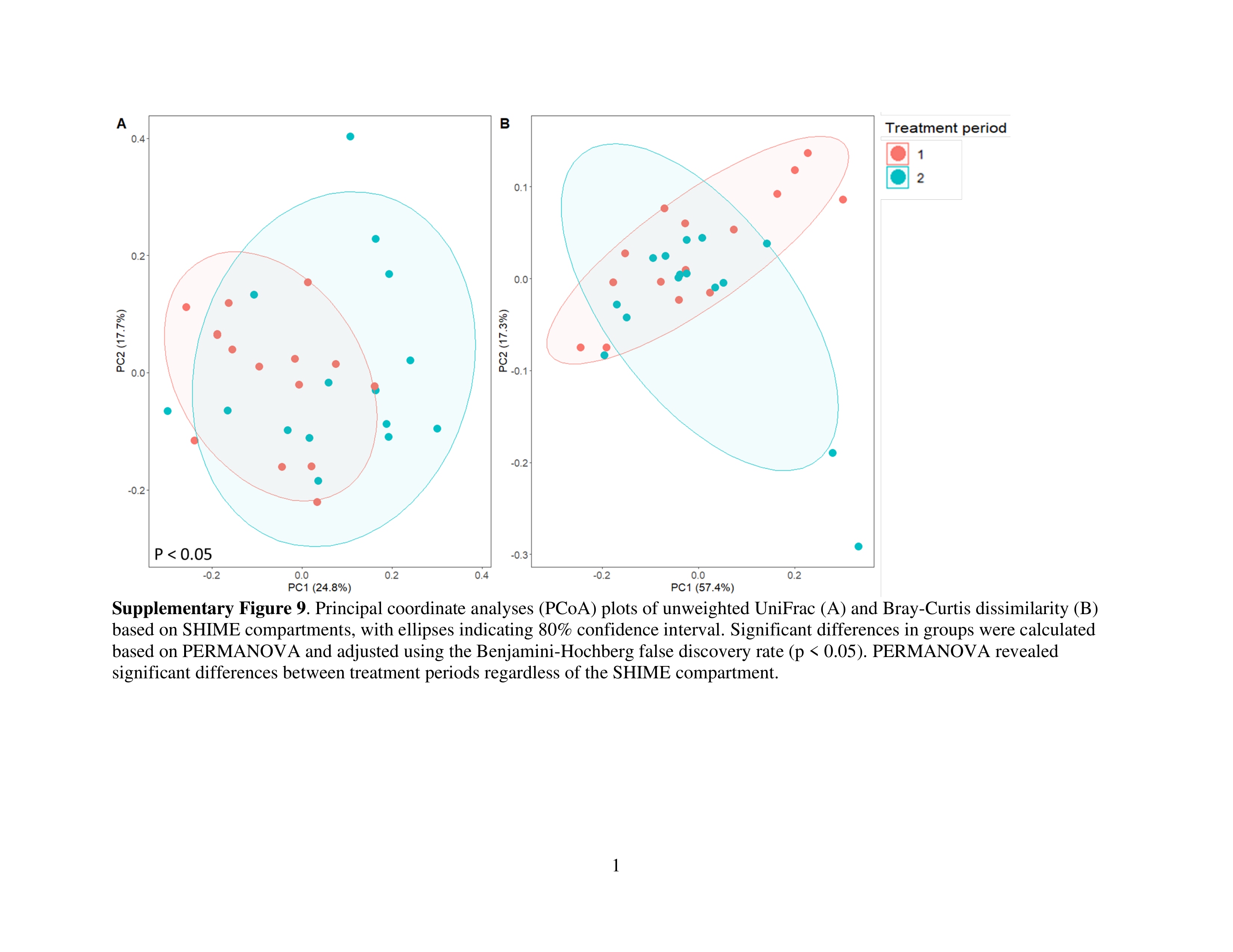

Supplement: Supplementary file 10 [file Image_9.JPEG]
